# Carbon nanotube filler enhances incinerated thermoplastics-induced cytotoxicity and metabolic disruption in vitro

**DOI:** 10.1186/s12989-020-00371-1

**Published:** 2020-08-12

**Authors:** Jayme P. Coyle, Raymond C. Derk, Tiffany G. Kornberg, Dilpreet Singh, Jake Jensen, Sherri Friend, Robert Mercer, Todd A. Stueckle, Philip Demokritou, Yon Rojanasakul, Liying W. Rojanasakul

**Affiliations:** 1 Health Effects Laboratory Division, National Institute for Occupational Safety and Health, Morgantown, WV USA; 2 Department of Pharmaceutical Sciences, West Virginia University, Morgantown, WV USA; 3 Department of Environmental Health, Harvard University, Boston, MA USA

**Keywords:** Incinerated thermoplastics, Nano-enabled composites, Polycyclic aromatic hydrocarbons, In vitro, Cytotoxicity

## Abstract

**Background:**

Engineered nanomaterials are increasingly being incorporated into synthetic materials as fillers and additives. The potential pathological effects of end-of-lifecycle recycling and disposal of virgin and nano-enabled composites have not been adequately addressed, particularly following incineration. The current investigation aims to characterize the cytotoxicity of incinerated virgin thermoplastics vs. incinerated nano-enabled thermoplastic composites on two in vitro pulmonary models. Ultrafine particles released from thermally decomposed virgin polycarbonate or polyurethane, and their carbon nanotube (CNT)-enabled composites were collected and used for acute in vitro exposure to primary human small airway epithelial cell (pSAEC) and human bronchial epithelial cell (Beas-2B) models. Post-exposure, both cell lines were assessed for cytotoxicity, proliferative capacity, intracellular ROS generation, genotoxicity, and mitochondrial membrane potential.

**Results:**

The treated Beas-2B cells demonstrated significant dose-dependent cellular responses, as well as parent matrix-dependent and CNT-dependent sensitivity. Cytotoxicity, enhancement in reactive oxygen species, and dissipation of ΔΨm caused by incinerated polycarbonate were significantly more potent than polyurethane analogues, and CNT filler enhanced the cellular responses compared to the incinerated parent particles. Such effects observed in Beas-2B were generally higher in magnitude compared to pSAEC at treatments examined, which was likely attributable to differences in respective lung cell types.

**Conclusions:**

Whilst the effect of the treatments on the distal respiratory airway epithelia remains limited in interpretation, the current in vitro respiratory bronchial epithelia model demonstrated profound sensitivity to the test particles at depositional doses relevant for occupational cohorts.

## Background

Thermoplastics, such as polycarbonate and polyurethane, are ubiquitous in the manufacture of commercial and consumer products due to their relative low cost, optical properties, and mechanical strength. Polycarbonate (PC) is used in automotive parts, construction materials, optical and medical devices, circuitry, and food and beverage packaging. Polyurethane (PU) is used in the automotive industry, high-pressure applications, and consumer products [1–3].

The scope of application in industrial and commercial products for both PC and PU is constantly expanding as new types of composites enabled with carbon nanotube (CNT) are being developed [4, 5], particularly for polycarbonate-CNT (PC-CNT) composites [6]. PC-CNT composites offer favorable attributes, including enhanced mechanical hardness, elastic modulus [7], tensile strength [8], and electrical conductivity [9] compared to parent polycarbonate matrices. The viscoelectric properties of PC-multiwalled CNT composites indicate alterations in the temperature-dependent melting behavior of PC [10], allowing these nano-enabled composites (NECs) to retain hardness over the duration of composite life even in the presence of thermal cycling [11]. PU-CNT composites also have superior physiochemical and mechanical properties compared to parent PU matrices [12, 13], increasing NEC use in commercial and industrial settings. Inclusion of novel NEC thermoplastics in commercial and consumer products can lead to potential exposures throughout the product’s lifecycle, including NEC particle release during production, fabrication, and use [14, 15] or disposal via incineration [16].

Of the 34.4 million tons of plastics disposed through the municipal solid waste (MSW) stream in the U.S., 5.34 million tons were incinerated for energy recovery [17]. Ever-increasing average tipping fees and decreasing number of operating landfills [18] suggest an increase in MSW being diverted for combustion for energy recovery in the future. Incineration of plastic waste in general results in the formation of volatile organic chemicals (VOCs) in both fly ash and flue gas streams [19, 20]. Though specific types of VOCs generated depends on temperature of combustion, common MSW incinerators (600–950 °C) predominantly generate low- and high-molecular weight polycyclic aromatic hydrocarbons (PAHs) [21–25] through catalytic secondary cyclization [26, 27]. The extent of catalysis depends on the presence and composition of engineered nanomaterial, leading to enhanced PAH profiles in nanomaterial-rich wastes [28]. The incinerated thermoplastics used in the present study, generated by simulated combustion in the Harvard INEX system, were previously shown to contain detectable amounts of PAHs within the aerosol whose concentrations increased for nano-enabled PU and PC compared to the virgin ones [29].

The exposure to, and potential health effects of, aerosol produced from advanced material NEC combustion is unknown, partially due to the lack of exposure data. Several epidemiological investigations have examined the associations between fine particulate with PAHs generated from diesel exhaust [30] or from the general urban environment [31–33] with pathological outcomes. PAHs have been purported as an etiological cause of pulmonary carcinogenicity [34] and are traditionally associated with the toxicodynamic properties of diesel exhaust particles (DEP), predominantly through organic extraction studies [35–38].

The potential public health risk of exposure to combustion aerosol particles of novel NECs remains under-researched. The present investigation aims to characterize the potential cytotoxic effects to the airway epithelia of incinerated virgin thermoplastics and incinerated NEC (iNEC) thermoplastic exposure in two histologically distinct regions of the human airway. The bronchus, modeled using the Beas-2B epithelial cell line, is integral for the pathogenesis of asthma and airway resistance [39], especially pathology induced by combustion-derived particulate matter [40]. The distal lung, modeled using primary small airway epithelial cells (pSAECs), is an important region for gas exchange. This investigation augments previous finding using the pSAEC model [41]. Subsequently, partitioning of PAH- versus particle-mediated effects was examined by behavioral measures, particularly since PAHs are implicated as contributory to the overall toxicity of combusted carbonaceous particulate including the iNECs.

## Results

### Particle characterization

**Fig. 1:**
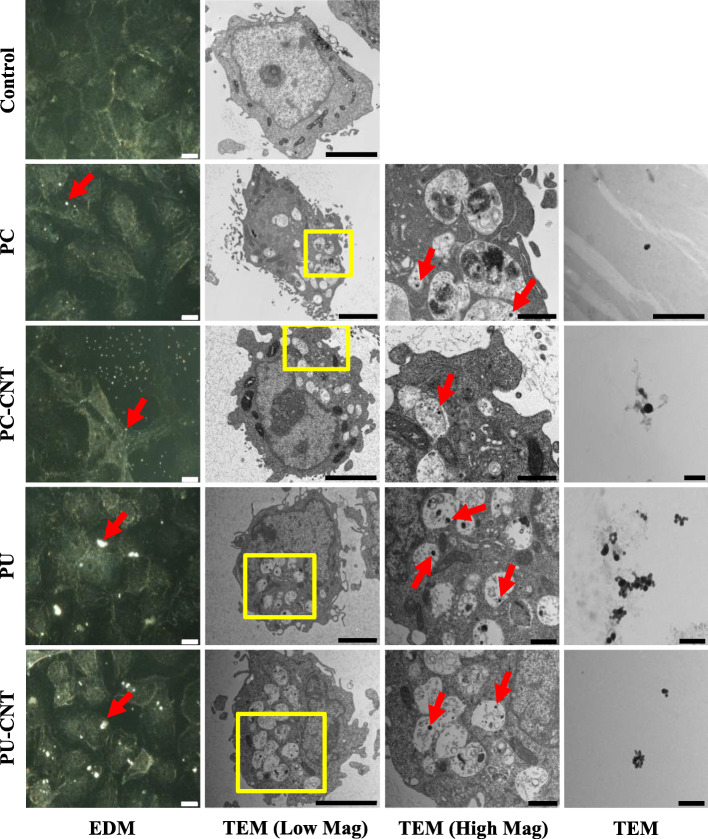
Microscopic assessment of Beas-2B cells treated with incinerated thermoplastics. Beas-2B treated with 0.6 μg/cm^2^ or 1.2 μg/cm^2^ incinerated for 48 h were visualized via enhanced darkfield microscopy (EDM) or transmission electron microscopy (TEM), respectively. Particles in suspension were also visualized by TEM for comparison against structures identified in Beas-2B cells. Incinerated thermoplastics visualized by EDM are identified by a bright spectral signature (Magnification: 60X, scale bar = 10 μm). Cells treated with incinerated thermoplastics were visualized under low magnification (Scale Bar = 4 μm) and high magnification (Yellow Box denotes region of high magnification, Scale Bar = 1 μm). Particle dispersed in water were prepared and visualized for TEM (Scale Bar = 1 μm). Endocytosed particles are identified by red arrows in EDM and high magnification TEM images

Stock particles diluted to 1 mg/mL in dH_2_O were sonicated for electron microscopy analysis for later comparison to intracellularly localized structures in vitro models. Morphological assessment of incinerated thermoplastics by scanning electron microscopy (SEM) and transmission electron microscopy (TEM) showed electron-dense particles (Fig. 1). EDX-assisted elemental compositional analysis of these particles yielded a carbonaceous signature, indicative of PC and PU. iNECs had additional signatures of aluminum and iron (Fig. S1), consistent with associated trace metals found in the CNTs used for composite formulation [29]. We did not attempt to further affirm the presence of nanofiller release in particle suspensions, as this was previously performed [42].

Particle hydrodynamic diameters and zeta potentials were determined using DLS (Table 1). Agglomerate hydrodynamic diameters of test particles ranged from 417 nm to 1208 nm with polydispersity indices (PDI) lower than 0.400, indicating good dispersion. Based on these colloidal characteristics, the volume-weighted hydrodynamic diameter of each particle, as well as depositional efficiency [Administered dose translating to deposited dose], were calculated from the Harvard Distorted Grid (DG) model. Particle volume-weighted diameters were generally smaller than incinerated virgin thermoplastics, necessitating higher administered dose loadings for iNECs to attain comparable deposited doses to virgin counterparts. Depositional doses tended to be higher in airway epithelial growth medium (AEGM) than comparable doses in small airway epithelial growth medium (SAGM), requiring higher thermoplastic loadings in SAGM for treating pSAECs with an analogous dose as Beas-2Bs (Table 2). Given the discrepancy in depositional efficiency, we utilized modeled depositional dose as the exposure metric rather than administered dose.

**Table 1:** Incinerated Thermoplastic Colloidal Characteristics - DLS

Particle	Medium	d_(H, Z-avg)_ (nm)	PDI	ζ (mV)	pH	σ (mS/cm)
PC	dH_2_O	867	0.200	−29.8	6.13	0.012
	AEGM	1208	0.140	−9.73	7.41	12.3
	SAGM	801	0.372	−12.7	7.48	12.2
PC-CNT	dH_2_O	541	0.048	−35.5	6.31	0.010
	AEGM	701	0.175	−9.85	7.42	12.2
	SAGM	587	0.206	−12.3	7.48	11.4
PU	dH_2_O	836	0.209	−6.97	5.34	0.014
	AEGM	724	0.284	−10.3	7.48	12.1
	SAGM	579	0.371	−10.7	7.50	11.6
PU-CNT	dH_2_O	603	0.233	−7.47	5.31	0.015
	AEGM	559	0.375	−9.21	7.53	12.1
	SAGM	417	0.305	−11	7.52	11.4

Incinerated thermoplastics were diluted to 0.1 mg/mL in respective medium and assessed for intensity-based hydrodynamic diameter [d_(H, Z-avg)_], polydispersity index (PDI), zeta potential (ζ), suspension pH, and suspension conductivity (σ)

**Table 2:** Incinerated Thermoplastic Colloidal Characteristics - Modeling

Particle	Medium	ρ_agg_ (g/cm^3^)	d_(Vol-weighted)_ (nm)	Deposition Efficiency
PC	AEGM	1.023	1255	18.6%
	SAGM	1.024	1288	17.4%
PC-CNT	AEGM	1.023	976	10.8%
	SAGM	1.028	792	8.2%
PU	AEGM	1.021	1092	21.8%
	SAGM	1.025	747	7.1%
PU-CNT	AEGM	1.020	601	9.3%
	SAGM	1.020	551	2.1%

DLS measurements in AEGM and SAGM were combined with aggregate density (ρ_agg_) derived from the volumetric centrifugation method, and volume-weighted hydrodynamic diameter (d_(Vol-weighted)_) in the Distorted Grid Model to derive the depositional efficiency of particle sedimentation into the in vitro cellular microenvironment averaged over a 72 h exposure

### Microscopy

**Fig. 2:**
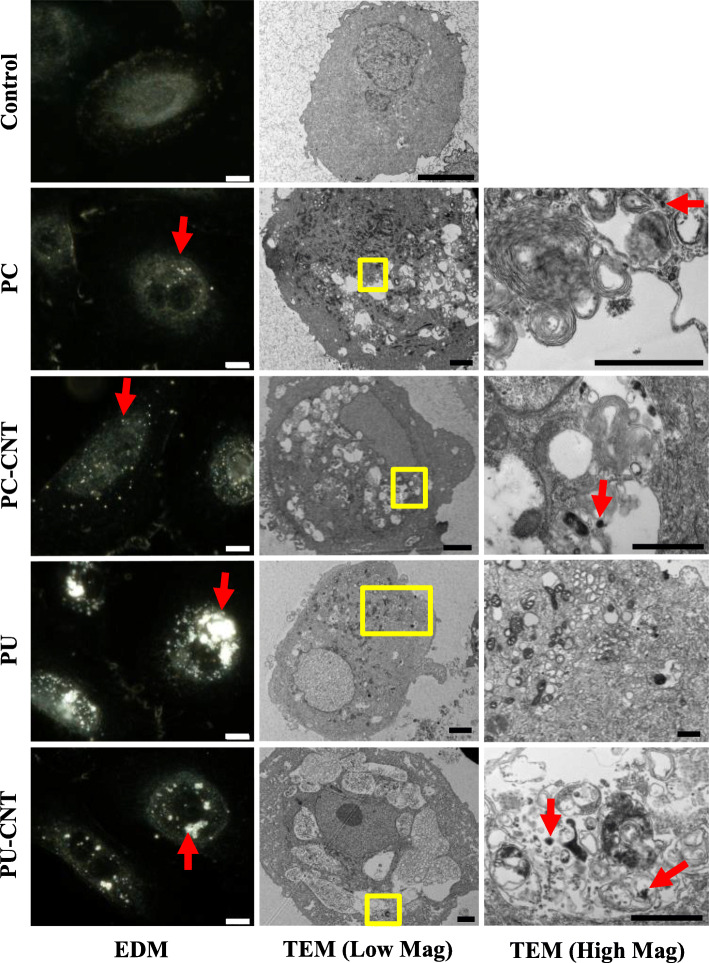
Microscopic assessment of pSAECs treated with incinerated thermoplastics. pSAECs treated with 0.6 μg/cm^2^ or 1.2 μg/cm^2^ incinerated for 48 h were visualized via enhanced darkfield microscopy (EDM) or transmission electron microscopy (TEM), respectively. Particles in suspension were also visualized by TEM (Fig. 1) for comparison against structures identified in pSAECs. Incinerated thermoplastics visualized by EDM are identified by a bright spectral signature (Magnification: 60X, scale bar = 10 μm). Cells treated with incinerated thermoplastics were visualized under low magnification (Scale Bar = 4 μm) and high magnification (Yellow Box denotes region of high magnification, Scale Bar = 1 μm). Endocytosed particles are identified by Red arrows

Stock particles diluted to 1 mg/mL in dH_2_O were sonicated and subsequently diluted in AEGM and SAGM for in vitro model exposure. Macroscopic localization of the particles showed cell-associated light-scattering diffraction signatures consistent with those of particle-only suspensions (Fig. S2), indicating nano-scale particles reached the cellular microenvironment. TEM examination of intracellular membrane-bound vesicles affirmed the presence of intracellularly-localized test particles (Fig. 1 and Fig. 2). PU/PU-CNT tended to associate with cells to a larger extent than PC/PC-CNT analogues at comparable deposited doses in both cell types, though we did not account for detachment of particle-laden cells after the 48-h treatment.

### Cytotoxicity

#### Beas-2B cell model

**Fig. 3:**
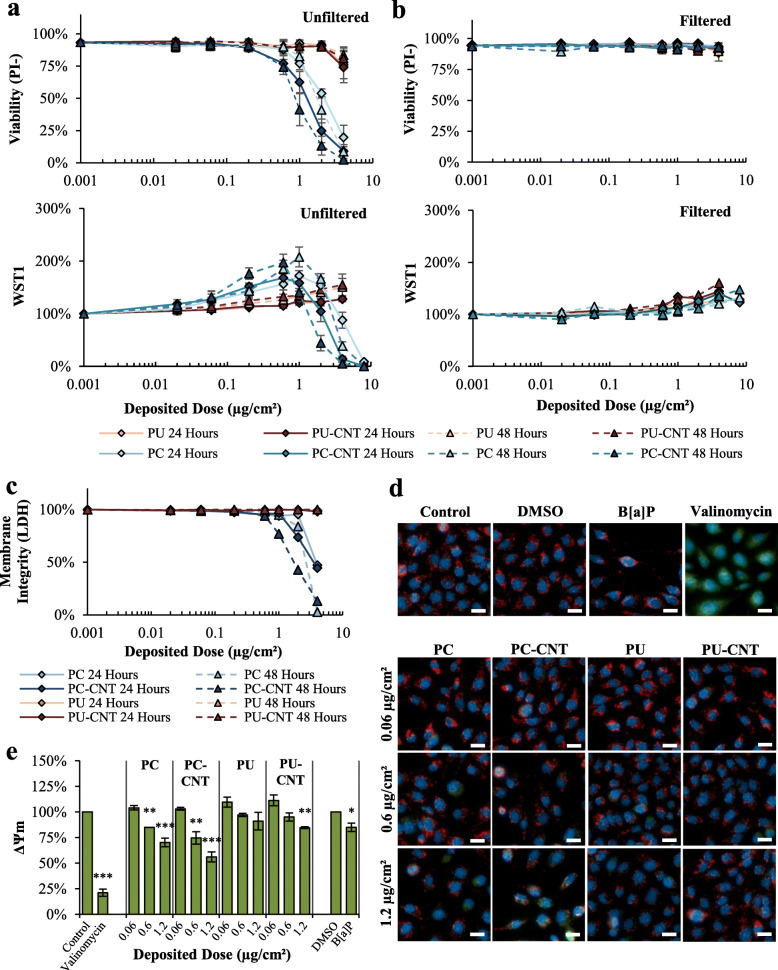
Cytotoxicity assessment of incinerated thermoplastics in Beas-2B Cells. a Dose-response curves to graded depositional doses of incinerated thermoplastics reported by live-cell imaging and WST1 reduction for particle containing suspensions. **b** Analogous dose-responses via live-cell imaging and WST1, except particle suspensions were filtered through a 0.2 μm pore to devoid treatments of particles, leaving only particle- associated leachables in AEGM. **c** Interference-adjusted LDH release 24 and 48 h after exposure to depositional doses used in live-cell imaging assessments. **d-e** Representative images of JC-1-reported ΔΨm measurement 24 h after exposure; scale bars = 20 μm. As a comparison, 0.5 μM B[a]P served as a representative PAH to compare to particle treatments; 10 μM valinomycin served as a positive control for ΔΨm dissipation. **e** Quantitative metrics of images analyzed are presented with statistical comparisons. Point estimates are the arithmetic mean of 2–4 independent experiments; error bars indicate standard error of the mean (SEM); *p < 0.05, **p < 0.01, ***p < 0.001 compared to respective controls. Images taken at 40X

Assessment via propidium-iodide adjusted for cell number (PI-) and WST1 metabolism (Fig. 3a) showed a depositional dose-dependent cytotoxicity of PC/PC-CNT above 1.0 μg/cm^2^ at 24- and 48-h post-treatment. Neither PU nor PU-CNT caused cytotoxicity detected by WST1 or PI-. The low-dose profile between the WST and PI- measures was markedly different for PC and PC-CNT. Treatment with 0.06–0.6 μg/cm^2^ PC/PC-CNT did not result in appreciable reductions in PI--reported cytotoxicity compared to controls but caused paradoxical hyper-stimulatory WST1 metabolism. Characteristic reductions in WST1 metabolism were observed beginning with 1.0 μg/cm^2^, in concordance with observed dose-dependent cell viability reduction reported by PI- assessment. The low-dose profiles were also significantly different between PC and PC-CNT: irrespective of assessment by PI- or WST1 or ED_50_ (Table 3), PC-CNT was significantly more potent (1.7–2.0-fold; *p* < 0.001) compared to PC at 24- and 48-h post-treatment. ED_50_ values derived from WST1 were approximately 2–3-fold higher than comparative PI-derived values (Table 3). Of note, potency differences between PC and PC-CNT were not apparent when depositional efficiency between particles was not factored (Fig. S3).

**Table 3:** Beas-2B Cytotoxicity Summarization

Filter Status	Time	Treatment	ED_50_ (μg/cm^2^)		Potency ED_50P_/ED_50CNT_	
			PI-	WST1	PI-	WST1
**Unfiltered**	24	PC	2.28	5.34	1.70***	1.92***
	24	PC-CNT	1.34	2.78		
	24	PU	> 4.0	> 4.0	N.D.	N.D.
	24	PU-CNT	> 4.0	> 4.0		
	48	PC	1.90	3.52	1.98***	1.86***
	48	PC-CNT	0.96	1.89		
	48	PU	> 4.0	> 4.0	N.D.	N.D.
	48	PU-CNT	> 4.0	> 4.0		
**Filtered**	24	PC	> 4.0	> 8.0	N.D.	N.D.
	24	PC-CNT	> 4.0	> 8.0		
	24	PU	> 4.0	> 4.0	N.D.	N.D.
	24	PU-CNT	> 4.0	> 4.0		
	48	PC	> 4.0	> 8.0	N.D.	N.D.
	48	PC-CNT	> 4.0	> 8.0		
	48	PU	> 4.0	> 4.0	N.D.	N.D.
	48	PU-CNT	> 4.0	> 4.0		

PI- – Cytotoxicity derived from live-cell imaging

WST1 – Cytotoxicity derived from the WST1 method

ED_50_ – Modeled deposited dose causing 50% of reported cytotoxicity compared to untreated controls

Potency ED_50p_/ED_50CNT_ – Relative deposited dose causing 50% of cytotoxicity among pristine incinerated thermoplastic (ED_50p_) compared to CNT-containing analog (ED_50CNT_) at the same time point. Potency comparisons across time points were not made

N.D. ED_50p_/ED_50CNT_ Potency value could not be determined

***p < 0.001 of compared to pristine incinerated thermoplastic at the same time point

To identify the contribution of particle-associated soluble compounds to the cytotoxicity of incinerated particles, thermoplastic preparations were clarified through a 0.2 μm PES filter prior to treatment. Filtration completely abrogated cytotoxicity (Fig. 3b; Table 3), while retaining minimal stimulatory WST1 metabolism, albeit with a substantially reduced maximal response. PC/PC-CNT showed detectable dose-dependent adjusted LDH release above 1 μg/cm^2^ at 24 and 48 h, while PU/PU-CNT showed no LDH release (Fig. 3c), thus confirming PI- measures.

To evaluate mitochondrial membrane potential (ΔΨ_m_), JC-1 ratiometric analysis was measured on three deposited doses: a non-cytotoxic dose (0.06 μg/cm^2^), the lowest observable deposited dose observed to induce significant cytotoxicity in PC-CNT-treated Beas-2B cells (0.6 μg/cm^2^), and a significantly cytotoxic dose for both PC and PC-CNT (1.2 μg/cm^2^). In accordance with PI-, the high dose of PC-CNT caused ΔΨ_m_ depolarization, as did Beas-2B treated with 0.6 μg/cm^2^ (Fig. 3d-e) that did not otherwise show appreciable cytotoxicity. PU-CNT caused significant ΔΨ_m_ depolarization only at the 1.2 μg/cm^2^ deposited dose. ΔΨm depolarization was also assessed using a fluorescent-reading plate reader. The trends were conserved between the two reading modalities, though the plate reader tended to overestimate ΔΨm depolarization (Fig. S4).

#### pSAE cell model

**Fig. 4:**
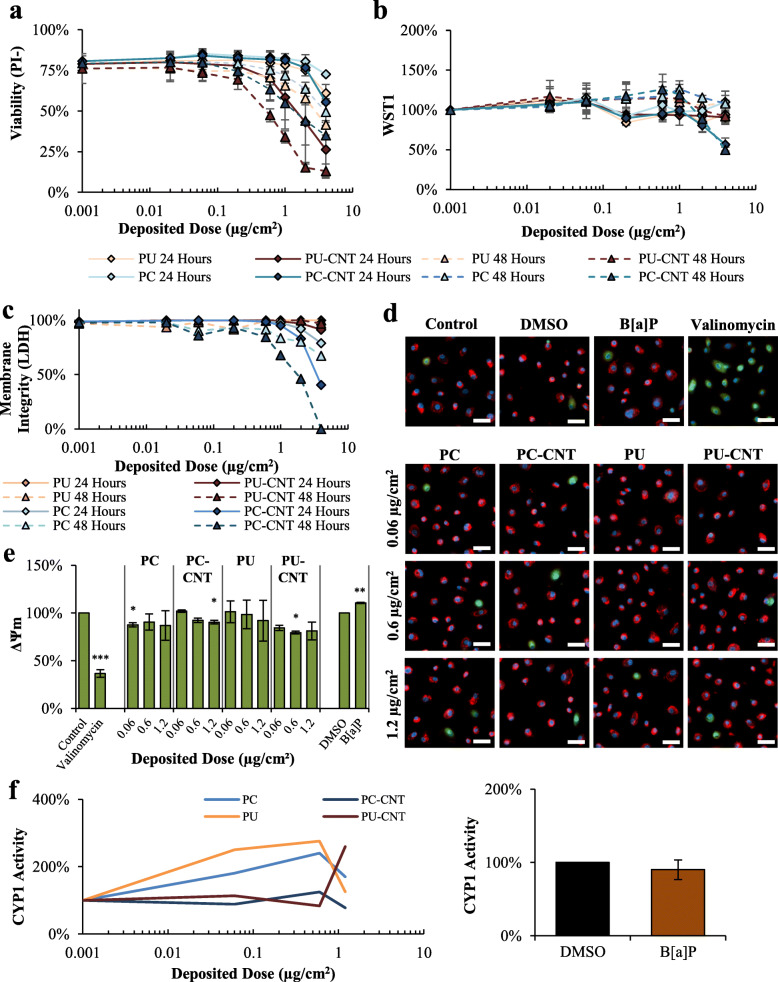
Cytotoxicity assessment of incinerated thermoplastics in pSAECs. **a-b** Dose-response curves to graded depositional doses of incinerated thermoplastics reported by live-cell imaging and WST1 reduction for particle containing suspensions. **c** Interference-adjusted LDH release 24 and 48 h after exposure to depositional doses used in live-cell imaging assessments. **d-e** Representative images of JC-1-reported ΔΨm measurement 24 h after exposure; scale bars = 50 μm. As a comparison, 0.5 μM B[a]P served as a representative PAH to compare to particle treatments; 10 μM valinomycin served as a positive control for ΔΨm dissipation. **e** Quantitative metrics of images analyzed are presented with statistical comparisons. **f-g** pSAECs were assessed for CYP1 activity 48 h post exposure to incinerated thermoplastics and 0.5 μM B[a]P. Point estimates are the arithmetic mean of 2 independent experiments; error bars indicate standard error of the mean (SEM); *p < 0.05, **p < 0.01, compared to respective controls. Images taken at 20X

Assessment via PI+CN and WST1 metabolism demonstrated significant dose-dependent cytotoxicity due to PC/PC-CNT and coincided with increases in LDH membrane permeability (Fig. 4a-c). Compared to Beas-2B cells, particle-induced WST1 hyperstimulatory metabolism in pSAECs was blunted, remaining below 130% of controls (Fig. 4b). Measurement via PI- also reported dose-dependent cytotoxicity of PU/PU-CNT that was not observed by WST1 assessments. Cytotoxic potency values derived from WST1 metabolism and PI- are shown in Table 4. The discrepancy between PI- versus WST1 was determined to be false positivity in live-cell imaging due to co-localization of cellular nuclei and fluorescently-active perinuclear aggregates of endocytosed PU/PU-CNT. pSAECs treated with 1.2 μg/cm^2^ of PC-CNT and PU-CNT demonstrated significant reductions in ΔΨ_m_ 24 h post exposure (Fig. 4d-e). B[a] P alone paradoxically increased pSAEC ΔΨ_m_ compared to DMSO-treated controls.

**Table 4:** pSAEC Cytotoxicity Summarization

Filter Status	Time	Treatment	ED_**50**_ (μg/cm^**2**^)		ED_**50P**_/ED_**50CNT**_ Potency	
			PI-	WST1	PI-	WST1
**Unfiltered**	24	PC	> 4.0	> 4.0	N.D.	N.D.
	24	PC-CNT	> 4.0	> 4.0		
	24	PU	> 4.0	> 4.0	N.D.	N.D.
	24	PU-CNT	2.21	> 4.0		
	48	PC	> 4.0	> 4.0	N.D.	N.D.
	48	PC-CNT	2.73	3.49		
	48	PU	> 4.0	> 4.0	N.D.	N.D.
	48	PU-CNT	0.83	> 4.0		

PI- – Cytotoxicity derived from live-cell imaging

WST1 – Cytotoxicity derived from the WST1 method

ED_50_ – Modeled deposited dose causing 50% of reported cytotoxicity compared to untreated controls

Potency ED_50p_/ED_50CNT_ – Relative deposited dose causing 50% of cytotoxicity among pristine incinerated thermoplastic (ED_50p_) compared to CNT-containing analog (ED_50CNT_) at the same time point. Potency comparisons across time points were not made

N.D. Potency ED_50p_/ED_50CNT_ value could not be determined as ED_50_ value for one or both groups exceeded highest concentration tested. None of the filtered preparations caused significant cytotoxicity in pSAECs

### CYP1 metabolism and WST1

**Fig. 5:**
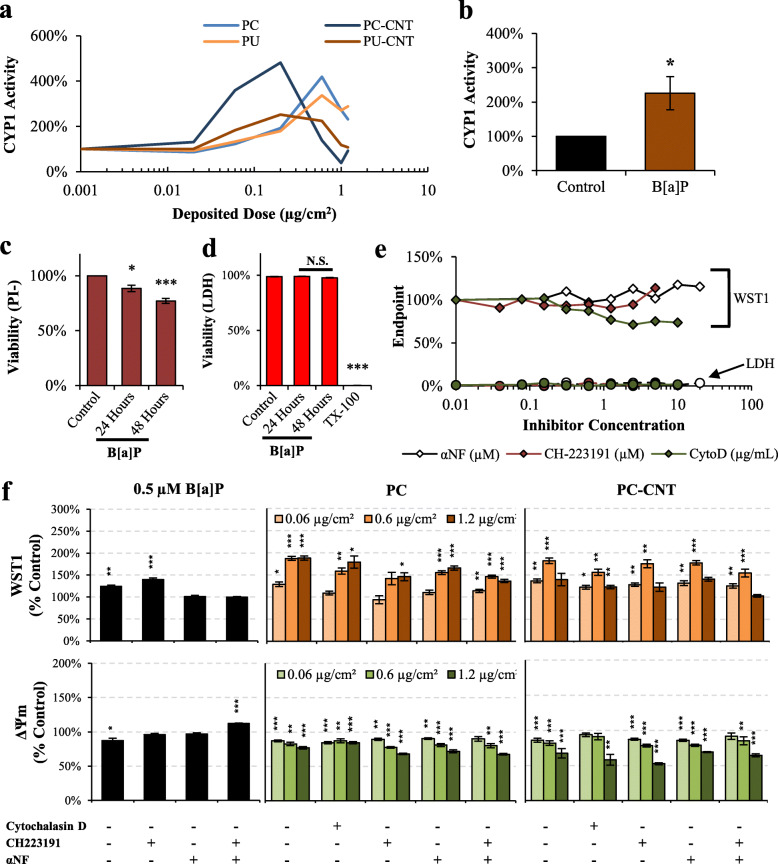
Characterization of AhR-associated effects in Beas-2B cells. **a-b** CYP1 activity 48 h post exposure to incinerated thermoplastics and 0.5 μM B[a]P. **c-d** B[a]P cytotoxicity assessment were conducted using live-cell imaging and membrane permeability (LDH). **e** Beas-2B cells were treated with graded concentrations of cytochalasin D (CytoD), CH223191, and αNF and assessed for proliferation (WST1; Diamonds) and membrane permeability (LDH; Circles) for co-exposure dose selection. **f** B[a]P- and incinerated thermoplastic-treated cells were co-incubated with 0.5 μg/mL CytoD, 5 μM CH223191, 10 μM αNF, CH223191 and αNF simultaneously, followed by assessment for WST1 metabolism and ΔΨm. Control bars are not displayed as all data points are normalized to respective controls designated as 100%. Point estimates are the arithmetic mean of 3–4 independent experiments; error bars indicate standard error of the mean (SEM); *p < 0.05, **p < 0.01, ***p < 0.001 compared to respective controls

It is well known that incineration of thermoplastic matrices produces an array of low- and high-molecular weight PAHs [19, 20], as was confirmed in a previous investigation using the simulated combustion system used to generate the particles used in the current investigation [29]. AhR-mediated CYP1 bioactivation by B[a]P has been shown to cause stable DNA adduct formation in B[a]P-treated Beas-2B cells, leading to genotoxicity and mutagenesis [43]. To ascertain bioavailability of particle-associated PAHs, CYP1 activity was measured as a proxy of AhR activation and subsequent transcriptional machinery for xenobiotic responsive element (XRE). All thermoplastics caused enhanced CYP1 activity at sub-cytotoxic doses, with the largest maximal effect found in Beas-2B treated with PC and PC-CNT peaking at 0.6 μg/cm^2^ (420% of controls) and 0.2 μg/cm^2^ (480% of controls), respectively. PU and PU-CNT elicited induction of CYP1 activity, albeit with lower effective maxima compared to PC/PC-CNT analogues (Fig. 5a). CYP1 activity in pSAECs treated with the test particles was enhanced, except for PC-CNT that remained approximately at control levels (Fig. 4f).

B[a]P treatment for 48 h was effective in inducing significant CYP1 activity in Beas-2B cells (Fig. 5b), even though there was no increase in CYP1 metabolism in pSAECs (Fig. 4g). These results suggest incinerated thermoplastic particles contained bioavailable PAHs that translated to enhanced xenobiotic response metabolic activity in Beas-2B cells in congruence with previous observations [44]. B[a]P also caused a significant reduction in PI- viability (Fig. 5c). Since the integrated PI- measure used to evaluate cytotoxicity accounts for both proliferation (cell number) and membrane permeability (PI positivity), the relative contribution of membrane permeability to the overall PI- estimate was not significantly different than untreated controls (3–5% PI+), indicating B[a]P cytotoxicity was driven by inhibition of cellular proliferation, rather than lytic cytotoxicity. This interpretation was verified by the lack of extracellular LDH post-exposure of B[a]P (Fig. 5d) in accordance with previous findings [45, 46].

To examine whether hyperstimulatory WST1 metabolism was associated with CYP1 metabolic competency, we employed the CYP1/AhR inhibitor αNF [47] and the specific AhR inhibitor CH223191 [48] to assess their effects on B[a]P- and PC/PC-CNT-treated Beas-2B cells. Neither αNF nor CH223191 caused cytotoxicity or inhibited proliferation at the tested concentrations (Fig. 5e). Pre-treatment with 10 μM αNF alone or in combination with 5 μM of the AhR inhibitor CH223191 reversed hyper-stimulated WST1 metabolism and attenuated B[a]P-induced ΔΨ_m_ depolarization (Fig. 5f). However, neither αNF alone nor in combination with CH223191 reversed PC- or PC-CNT-induced WST1 stimulatory metabolism or ΔΨ_m_ depolarization (Fig. 5f). As rescue was not attained through pharmacological inhibition, PC/PC-CNT particle induced cytotoxic effect in Beas-2B cell seems not to depend on PC/PC-CNT-associated PAHs. While the observation of WST1 hyperstimulation in the presence of PAH-associated exposures in Beas-2B is consistent with previous findings of diesel exhaust particle extract-exposed Beas-2B cells [35]. Despite such an observation, that study did attempt to elucidate the source or implications of hyperstimulated WST1 metabolism. To examine if particle uptake was necessary for inducing cytotoxic injury, we employed the actin inhibitor cytochalasin D. Cytochalasin D alone significantly inhibited Beas-2B proliferation, as reported by WST1 metabolism, without significant induction of membrane permeability (Fig. 5e) in accordance with previous findings [49]. Upon co-treatment, 0.5 μg/mL cytochalasin D only partially reversed PC/PC-CNT-induced ΔΨ_m_ depolarization (Fig. 5f).

### Cell cycle and genotoxicity

Since the current understanding presumes desorption of particle-adsorbed PAHs leads to genotoxicity [50, 51], we hypothesized particle-adsorbed PAHs would be bioavailable to cause activation of AhR-mediated XRE-associated machinery in Beas-2B cells, resulting in genotoxicity [52–55] and partially accounting for reductions in PI- observed in acute cytotoxicity assessment. Further, we examined proliferative behaviour of Beas-2B after treatmments in order to correlate particle-mediated effects to B[a]P, the archetypal PAH.

**Fig. 6:**
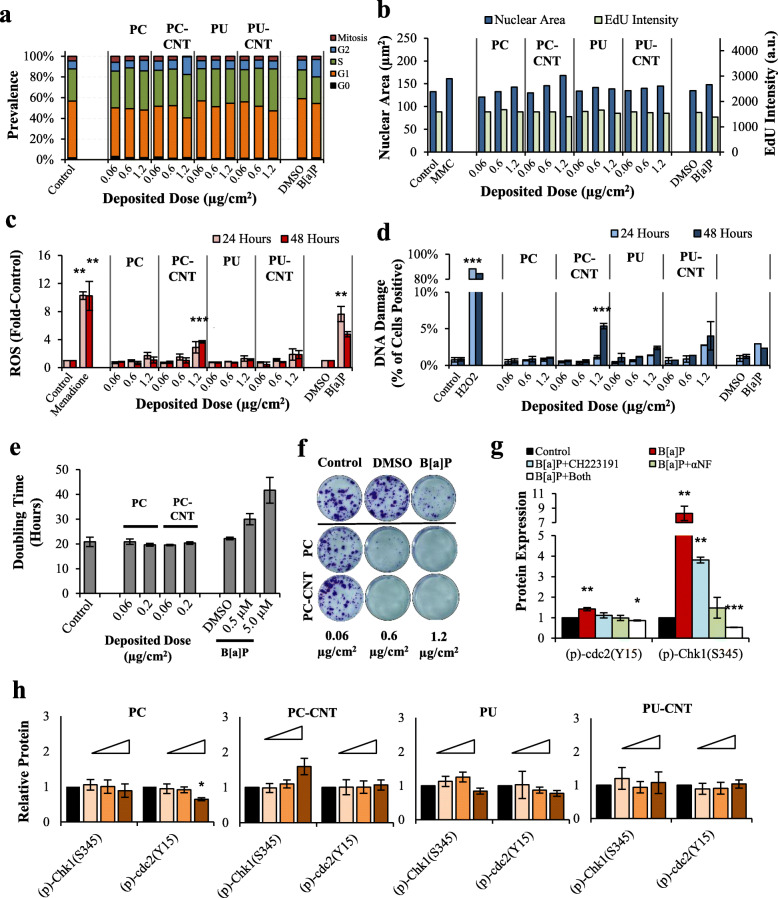
Cell cycle analysis and nuclear morphometry in Beas-2B cells. **a-b** Quantitative binning of cell cycle-specific phases and nuclear morphometry, including nuclear area and EdU uptake [in arbitrary units (a.u.)] analyzed from high-content screening 24-h post treatment. MMC = 0.76 ng/mL mitomycin C – a clastogen control. Results are from a single experiment. **c** Intracellular ROS was measured 24 and 48 h after treatment; 100 μM Menadione served as a positive control for ROS generation. B[a]P-induced intracellular ROS was significant at both time points - limited space precluded asterisk placement above the 48-h time point. **d** Cells treated for 24 and 48 h were stained for yH2AX; H_2_O_2_ served as a positive control. Results are presented as percent of cells positive for yH2AX out of the total cell population (> 1000/experiment). **e** Cells treated with PC/−CNT and B[a]P assessed for doubling time from growth curves from 3 independent experiments. **f** Clonogenic assay of Beas-2B cells treated for 3 days. **g** Western Blot analysis of B[a]P-treated cells for 24 h with and without inhibitors for AhR (Ch223191) or CYP1 (αNF). **h** Western Blot analysis of Beas-2B treated with 0.06, 0.6, and 1.2 μg/cm^2^ cells for 24 h; controls are solid black whilst concentrations are indicated by wedge where the 1.2 μg/cm^2^ treatment is represented by the thickest portion of the wedge. Point estimates for **d** and **f** are the arithmetic mean of 2–3 independent experiments; error bars indicate standard error of the mean (SEM); *p < 0.05, **p < 0.01, ***p < 0.001 compared to respective controls

Having affirmed dose-dependent CYP1 activity induction, indicating AhR mobilization, follow-up high-content screening showed perturbations in cell cycle phase distribution and nuclear morphometry due to B[a]P and the high dose of PC-CNT in asynchronous Beas-2B cells (Fig. 6). The 1.2 μg/cm^2^ dose PC-CNT caused a substantial reduction of mitotically active cells (phospho-Histone H3^Ser10^+), nuclear enlargement, and enhancement of G2- and S-phase prevalence (Fig. 6a-b). Despite enhancement of S-phase prevalence, EdU uptake, a proxy of DNA synthesis, was reduced. Accompanying cell cycle and morphological parameters, PC-CNT caused a significant increase in ROS (Fig. 6c) as well as γH2AX formation (Fig. 6d), the latter a marker of genotoxic double strand breaks [56, 57]. Perturbations of cell cycle kinetics were transient, as assessment of doubling time after subacute treatment suggested that neither PC nor PC-CNT reduced proliferative capacity between 0.06 μg/cm^2^ and 0.2 μg/cm^2^ (Fig. 6e), despite almost complete abrogation of clonogenic growth when exposed to > 0.6 μg/cm^2^ PC/−CNT (Fig. 6f). Among B[a]P-only treated Beas-2B cells, phosphorylation of Chk1 and cdc2 was reversible upon pre-treatment with 10 μM of the CYP1 inhibitor αNF (Fig. 6g), though PC/PC-CNT-treated Beas-2B cells did not exhibit inhibition of cell cycle kinetics as detected by proteomic analysis (Fig. 6h). A summary of endpoints for the test particles-treated Beas-2B cells are presented in Table 5. In pSAEC model, all test particles did not cause significant increases in intracellular ROS as reported by CellROX green, denoting treatments did not affect intracellular redox potential (Fig. S5). Additionally, neither PC/PC-CNT nor B[a]P induced significant γH2AX formation in pSAECs, while no conclusions could be drawn on PU/PU-CNT-treated cells since thermoplastic-associated non-specific fluorescence caused substantial false-positivity (Fig. S6).

**Table 5:** Summary of Measured Endpoints – Beas-2B

	Cytotoxicity				Genotoxicity					Mechanistic	
Agent	ΔΨm	LDH Release	WST1	Clonogenic Proliferation	yH2AX	(p)-Histone H3	Nuclear Area	EdU Uptake	Cell Cycle Checkpoint	ROS	CYP1
PC	↓	↑	↑↓*	↓	–	–	–	–	–	–	↑
PC-CNT	↓	↑	↑↓*	↓	↑	↓	↑	↓	–	↑	↑
PU	–	–	↑	N.D.	–	–	–	–	–	–	↑
PU-CNT	–	–	↑	N.D.	–	–	–	–	–	–	↑
B[a]P	↓	–	↑	↓	–	–	↑	↓	+	↑	↑

↓ Significant reduction at any tested dose; ↑ Significant increase at any tested dose. – No change at any dose. N.D. Not Determined. *Low depositional dose caused increased metabolism but reduced at higher concentrations

## Discussion

The results demonstrate aerosolized particulate matter from incinerated polycarbonate causes significant cytotoxicity and alterations in metabolic function dose-dependently in bronchial epithelial cells, but not in small airway epithelial cells. Addition of 3% CNT in the polycarbonate matrix significantly enhanced measured endpoints in bronchial epithelial cells. Conversely, aerosolized particulate matter of pristine and 0.1% w/w CNT-enabled polyurethane were not cytotoxic in both in vitro models.

Deposition of virgin and iNEC particles tested was confirmed using enhanced darkfield and electron microscopy, demonstrating not only deposition, but also endocytosis of all particles examined. Despite little apparent cytotoxicity or genotoxicity, PU/PU-CNT tended to accumulate more extensively in both cell types than PC analogues; the reasoning of which was not investigated in this work. Baulig et al. [58] noted a similar phenomenon when examining airborne particulate matter, DEP, and carbon black in a human bronchial epithelial model, thus attributing partially the underlying toxicodynamic effects to particle-specific physiochemical parameters independent from access to the intracellular space. Whether endocytosis represents a unified fate for combusted carbonaceous materials or the product of an in vitro particle-medium interaction, e.g., proteinaceous corona that facilitates endocytosis, has yet to be elucidated [59–61]. Since cytochalasin D co-treatment with PC/PC-CNT did not completely mitigate ΔΨ_m_ reductions, the contribution of partico-extracellular interactions as well as cytochalasin D-insensitive uptake, or a combination of uptake pathways [62], remains unknown in the current system. Thorugh filtration studies, the presence of particles, particularly PC/PC-CNT, were essential for eliciting the cytotoxicity and metabolic dispruption observed in bronchial cells.

As determined thorugh filtration studies and the use of B[a]P as a PAH control, the incinerated parent matrix largely determined cytotoxicity and metabolic disruption, while the incorporation of CNT exacerbated observable parent matrix effects. We do not expect direct contributory toxicity from intact CNTs as they have been shown to thermally decompose to CO_2_ at 800 °C [63–65], even when incorporated in thermoplastic matrices [42]. While contribution to toxicodynamic effects of CNT-associated impurities, such as iron and aluminum, cannot be discounted [66], this was not explored further in this investigation. For both metals, high doses, typically exceeding the particular doses utilized in this study, are required to induce cytotoxic endpoints [67, 68]. Byproducts of incineration, such as PAHs, provide a likely source of accounting for the enhanced cytotoxicity profile, particularly since incineration of iNECs utilized in this study significantly enhance the PAH profile of the aerosol particulates [29]. Despite enhancement in PAH generation associated with these particles, we did not observe PAH-isolated effects in either Beas-2B or pSAECs. Among Beas-2B cells, filtration studies resulted in only mild metabolic changes with no cytotoxicity, denoting PAH leaching into the medium did not contribute substantially to the observed effects in Beas-2B cells. To ascertain bioavailability of PAHs adsorbed to the particles, we co-treated PC/PC-CNT with αNF and/or CH223191. Despite reversal of the significant ΔΨ_m_ depolarization and WST1 metabolic changes among B[a]P-treated Beas-2B cells, both inhibitors were iineffective in reversing effects observed among PC/PC-CNT treatment groups, denoting the particles as the primary source of cytotoxic injury and metabolic changes.

Similar to cytotoxic endpoints, the primary particle of the incinerated thermoplastics was the primary contributor to genotoxicity and changes in cell cycle kinetics in Beas-2B cells. Neither PC/PC-CNT nor B[a]P induced γH2AX formation in pSAECs at the tested doses in this investigation. PU/PU-CNT were found to have interfered with assessment, leading to erroneous positivity dose-dependently, the source of which was not examined in the current investigation. The high-dose PC-CNT resulted in significant increases in γH2AX+ and decreases in phospho-Histone H3^Ser10^+ nuclei in Beas-2B cells. Similarly, nuclear enlargement was in similar magnitude as mitomycin c – an archetypal clastogen [69]. Taken together, the combination of increased γH2AX formation and reduced phospho-Histone H3^Ser10^+ have been shown as predictive multi-parameter phenotypes for classifying agents as clastogenic genotoxicants [70, 71]. In comparison, B[a]P, a known clastogen [52, 54, 55, 72], caused insignificant elevations with positive γH2AX nuclear localization in percent of cells as well as stark changes in nuclear area and EdU incorporation. The lack of significant genotoxicity, as measured by γH2AX localization, at the tested dose of 0.5 μM B[a]P was consistent with other reports [73, 74]. B[a]P caused modest increases in G2-phase and slight reductions in S-phase and mitotically active cells, largely in accordance with previous findings in the Beas-2B cell line [45, 75, 76]. These three studies relied on cell cycle determination using Gaussian-derived parameterization of DNA content from flow cytometric analyses that are sensitive to misclassification of early- and late-phase S cells to G0/G1 and G2/M, respectively [77], as well as the inability to discriminate G2 versus mitosis, unless specific staining methods are employed [78]. As such, the method employed to characterize cell cycle kinetics allows for a more complete cell cycle distribution than nuclei intensity-based methods.

Kinetic changes in cell cycle among B[a]P-treated cells were attributable to phosphorylation of Chk-1(S345) and cdc2(Y15) associated with cell cycle checkpoint activation [79–85], and were reversible upon pre-treatment with the CYP1 inhibitor αNF. These results indicate intact CYP1 metabolism as implicated in precipitating alterations in cell proliferation in accordance previous findings [86]. Conversely, PC/PC-CNT did not significantly alter phosphorylation of Chk-1(S345) and cdc2(Y15) or doubling time, despite being acutely cytotoxic at concentrations > 1.0 μg/cm^2^. The overall cytological effects of PC/PC-CNT occurred independently of PAH-associated effects in our model system, despite CYP1 activity induction. The intracellular concentration of bioavailable PAHs likely remained below the threshold required for inducing genotoxic insult among thermoplastic-treated Beas-2B cells, though this is difficult to ascertain given the heterogeneous PAH mixture of these samples and unmeasured quantity of particle-associated PAHs in the present system. However, a limitation of this study pertains to the nature of particle preparation; namely, aerosolized particles were collected and extracted from filters into an aqueous suspension for in vitro testing in a submerged system. Therefore, any contribution to PAH bioavailability by lung lining fluid [87] remains unknown as desorption in these fluids may enhance B[a]P bioavailability [88], and subsequently produce a PAH-mediated effect in vivo that we were unable to recapitulate. Overall, the same incinerated thermoplastics treated pSAECs showed a blunted cytotoxic response compared to the magnitude of that observed in Beas-2B, demonstrating cell-specific heterogeneity in sensitivity to incinerated thermoplastics to cytotoxicity. Likewise, metabolic heterogeneity between the pSAECs and Beas-2B was observed. CYP1 metabolism in pSAECs was observed for most iNECs, except by PC-CNT, while 0.5 μM B[a]P did not induce CYP1 activity. To address B[a]P, Chang et al. [89] has shown pSAECs demonstrate a blunted response to AhR ligands compared to Club cells, resulting in modest transcriptional upregulation of CYP1 isoforms upon treatment with B[a]P. Therefore, pSAECs are likely inherently less sensitive to PAH-induced CYP1 transcription and subsequent metabolism compared to bronchial cells; the results from the current investigation tend to support this notion on the one hand. On the other hand, iNECs induced robust CYP1 activity in pSAECs whose activity maxima were lower than Beas-2B cells, suggesting either the PAH threshold to induce metabolic induction in pSAECs was exceeded by iNECs or iNECs may cause metabolic induction independently of PAH-associated nuclear receptors, such as AhR.

Due to the substantial induction of WST1 metabolism by PC/PC-CNT, we employed several methods to delineate changes in metabolism, membrane permability, proliferation, and mitochondrial function to qualify the in vitro response observed. The discrepancy between WST1 and PI−/LDH was attributable to the hyper-stimulatory WST1 metabolism observed along the range of doses examined, whereas these treatment groups did not demonstrate enhanced proliferative capacity (See Cell Cycle and Genotoxicity Section). As such, the dose at which WST1 metabolism inflection occurs is above 1.0 μg/cm^2^, delineating the dose at which cytotoxicity outweighed hyperstimulatory metabolism. Since PI- method integrates both cell number as well as membrane permability, this method serves as an indicator of proliferative capacity and membrane permeability. WST1, by contrast, measures the rate of formazan metaboplism via reduction of an electron-coupling intermediary [90]. Recently, B[a]P has been shown to upregulate genes associated with the pentose phosphate pathway [91], leading to enhanced NADPH formation and WST1 metabolism [92]. While the prevailing interpretation of formazan metabolism centers around mitochondrial function, the authors counter such an interpretation under the circumstances of the current manuscript, especially since we observed significant reductions in ΔΨ_m_ among Beas-2B cells treated with both B[a]P and PC/PC-CNT. B[a]P and depositional doses > 0.2 μg/cm^2^ PC/PC-CNT likely resulted in enhanced NADPH formation in Beas-2B cells without overt cytotoxicity, leading to hyperstimulatory WST1 metabolism. Given the results of cell cycle analysis, enhanced WST1 could not be attributed to enhanced proliferation. PI- showed no discernible changes up to 0.6 μg/cm^2^ which was observed as the point of departure for PC-CNT for cytotoxicity. Therefore, interpretation relied on PI- as the primary representation of cytotoxicity, while WST1 likely served as a proxy of NADPH-associated metabolic induction.

The cytotoxicity potency differences of PC vs. PC-CNT would not be readily identifiable when regressing solely on administered dose. In effect, the results suggest, without ascribing dosing metrics based on differential deposition of each thermoplastic, characterization by administration dose underestimates the cytotoxic potency of both PC and PC-CNT. Further, interpretation of administrative doses does not capture the obvious potency differences between the two incinerated materials, thus, underscoring the importance of characterizing delivered dose in order to accurately realize comparative cytotoxic potency [93].

## Conclusions

In conclusion, all virgin and CNT-containing thermoplastics tested were to some extent endocytosed by the in vitro models of the human bronchus (Beas-2B) and the distal airway (pSAECs). In both cell lines, PU/PU-CNT proved highly endocytosed but not acutely cytotoxic. By contrast, PC/PC-CNT was acutely cytotoxic in both cell lines, while Beas-2B cells uniquely more sensitive than pSAECs. In Beas-2B cells, PC-CNT approximately 2-fold more cytotoxic than PC. Presuming the dose group of B[a]P utilized in this investigation serves as a surrogate for total PAHs, adsorbed PAHs contributed negligibly to the overall toxicodynamic effect acutely, at least among Beas-2B cells. Observation of PC and PC-CNT induced increased WST1 metabolism, and DNA damage in Beas-2B cells need further sub-chronic investigation to ascertain if exposure to these incinerated thermoplastics may pose a potential carcinogenic or asthmatic risk to human health, particularly in the upper airway.

## Methods

Methods described here have been abridged. For the complete details on the methods utilized in this investigation, please refer to the Additional fsile 2.

### Particle characterization and dosimetry

All incinerated virgin and NECs used in this study were provided by Dr. P. Demokritou’s lab at Harvard University following extraction from in-line filters. Generation of incinerated thermoplastic samples using the Harvard INEX system and particle extraction are described elsewhere [94–96]. Previous work using these materials fully characterized their physicochemical properties, including CNT loading and signature impurities [29, 42].

Particle deposition for thermoplastics was modeled using the Harvard Distorted Grid (DG) model as described in GM DeLoid, JM Cohen, G Pyrgiotakis and P Demokritou [97] to estimate average deposited dose up to 72 h post-treatment. Deposited modeling was conducted using MatLab v. R2017b (MathWorks, Inc., Natick, MA). Except for data derived from dynamic light scattering and deposited modeling, methodological approaches for ascertaining medium density, viscosity, and refractive index may be found within Table S1 and Supplemental Methods.

#### Hydrodynamic diameter by dynamic light scattering

The critical delivered sonication energy (DSE_cr_), which is the energy necessary to disperse 1 mg/mL of the particle suspension in dH_2_O was derived previously as 1066 J/mL for all thermoplastics [41]. Stock particle suspensions in dH_2_O were sonicated using a Cup Horn Sonicator (Sonics VibraCell VCX-750 with Cup-type Sonicator; Newton, CT) immediately prior to dilution to 0.1 mg/mL in dH_2_O or culture medium. A Zetasizer Nano ZS and DipCell for electrophoretic measurements (Malvern Instruments; Malvern, United Kingdom) was used to evaluate hydrodynamic diameter, zeta potential, and conductivity of particle suspensions. Suspension pH was measured with a standard pH meter (Accumet Model 50, Fisher Scientific). Dispersant parameters required for DLS measurements are included in Table S1.

#### Effective density

To assess effective density via the volumetric centrifugation method [97] using packed cell volume tubes (Techno Plastic Products, A.G., Trasadigen, Switzerland) and pellet volume measured manually using a PCV tube ruler (Techno Plastic Products, A.G.). The density of the parent material, packed pellet volume, and medium density were performed in triplicate and used to calculate the effective density (*ρ*_*EV*_) requisite for modeling.

#### Endotoxin content

All thermoplastics were screened for endotoxin content at a suspension concentration of 10 μg/mL in endotoxin-free water according to the NCL Method STE-1.1 [98], and were found to be below the detection limit of < 0.01 EU/mL via the LAL chromogenic method (Pierce Biotechnology, Inc.; Rockford, IL). In assessing endotoxin adsorption, 10 μg/mL particle suspensions were spiked with 0.05 EU/mL, which resulted in bound endotoxin ranging from 0 to 23% (Fig. S7), depending on the thermoplastic. The spike-in control of 0.05 EU/mL was chosen as it was the midpoint between the minimum and maximum concentrations of the high-sensitivity method utilized (0.01–0.1 EU/mL), allowing for estimation within the method’s dynamic range.

### Cell culture and treatment

Human bronchial epithelial (Beas-2B) cells were purchased from ATCC (CRL-9609, Manassas, VA), and cultured in complete airway epithelial growth medium (AEGM) purchased from PromoCell, GmbH (Heidelberg, Germany) supplemented with 10 ng/mL epidermal growth factor, 5 μg/mL insulin, 0.5 μg/mL hydrocortisone, 0.5 μg/mL epinephrine, 10 μg/mL transferrin, 0.1 ng/mL retinoic acid, 6.7 ng/mL triiodo-L-thyronine, and 4 μL/mL Bovine pituitary extract. Experimentation on Beas-2B cells was performed on cells passaged between a total of 7 and 14 times. Human primary small airway epithelial cells (pSAECs) were purchased from PromoCell and maintained in small airway epithelial growth medium (SAGM) supplemented with 10 ng/mL epidermal growth factor, 5 μg/mL insulin, 0.5 μg/mL hydrocortisone, 0.5 μg/mL epinephrine, 10 μg/mL transferrin, 0.1 ng/mL retinoic acid, 6.7 ng/mL triiodo-L-thyronine, 4 μL/mL Bovine pituitary extract, and 2.5 mg/mL fatty acid-free Bovine serum albumin from the same vendor. Experimentation on pSAECs was performed on cells passaged between 4 and 8 times. Cultures were maintained in a humidified 37 °C incubator (Fisher Scientific) under an atmosphere of 5% v/v CO_2_ and balance air.

Both cell types were seeded into microplates at sub-confluency as described for individual assays below. The culture medium was aspirated and replaced with fresh pre-warmed medium containing treatment particle at designated delivered doses, calculated as described above. 1.0 mg/mL stock solutions of all test particles were sonicated to immediately before serial dilution in growth medium. All dosing designations are reported as deposited dose (μg/cm^2^). For indicated experiments, cells were pre-incubated with an actin polymerization inhibitor cytochalasin D (CytoD; Millipore-Sigma), aryl hydrocarbon receptor inhibitor CH223191 (Millipore-Sigma), or an aryl hydrocarbon receptor/CYP1 inhibitor alpha-naphthoflavone (αNF; Millipore-Sigma), for 1 hour before the incinerated thermoplastic treatments, and continued for the duration of incinerated thermoplastic exposure.

### Microscopy

To evaluate particle uptake in SAECs and Beas-2B cells, two methods of microscopic analysis were employed.

#### Enhanced Darkfield microscopy

Cells were seeded onto round laser-cut glass coverslips (Schott, A.G.; Jena, Germany) within 6-well tissue culture-treated microplates at a density of 150,000 cells per well. Two days later, cells were treated with 0.6 μg/cm^2^ of each thermoplastic in complete medium for 48 h, fixed in 4% formaldehyde in DPBS, and mounted on slides. Coverslips were then imaged at 60X using the CytoViva EDM system (CytoViva, Inc.; Auburn, AL).

#### Electron microscopy

Cells were seeded at a density of 150,000 cells per well in tissue culture-treated 6-well microplates. Cells were then treated for 48 h with 1.2 μg/cm^2^ incinerated thermoplastics, trypsinized with 0.25% Trypsin EDTA, washed twice with D-PBS, and fixed in Karnovsky’s fixative overnight as a cell suspension. Cells were pelleted, embedded in agarose, fixed, stained, and embedded in epon prior to sectioning. Particle preparations were imaged for SEM using the Hitachi S4800 field-emission scanning electron microscope (FESEM; Tokyo, Japan) with energy dispersive x-ray (EDX) generated qualitative elemental analysis (Bruker Nano, Berlin, Germany). Cell and particle preparations for TEM were imaged using the JEOL 1400 transmission electron microscope (Tokyo, Japan).

### Cytotoxicity, membrane permeability, and proliferation assessment

Five measures were employed to describe cytotoxicity, membrane permeability, and proliferative capacity: WST1, LDH, clonogenic assay, live cell imaging, and mitochondrial membrane potential. Except for the clonogenic assay, Beas-2B and pSAECs were plated at a density of 5000 and 2500 cells per well, respectively, in 96-well microplates 2 days prior to exposure. For the post-treatment proliferative capacity, cells were plated in 6-well microplates at a density of 50,000 cells per well 2 days prior to treatment. Benzo[a]pyrene (B[a]P; 0.5 μM) served as a PAH control and was purchased from Millipore-Sigma. All colorimetric endpoints were acquired using the SpectraMAX Plus 384 (Molecular Devices; San Jose, California) and SoftMax Pro v. 5.4.1 data acquisition software.

#### WST1 tetrazolium reduction

Cells were treated with indicated particle in growth medium for 24–48 h prior to assessment with WST1 (Millipore-Sigma, St. Louis, MO). After incinerated thermoplastic exposure an aliquot of supernatant abstracted into a clean clear-bottom microplate for LDH assessment (see below). The remaining culture medium was replaced with fresh medium, and the WST1 assay was performed according to the manufacturer’s instruction with a 2-h incubation period. WST1 formazan was quantitated at 450 nm with a reference wavelength of 650 nm. Treatment groups were normalized against medium-only controls, which were arbitrarily denoted as 100%.

#### Lactate dehydrogenase activity

The LDH assay was performed according to manufacturer’s instructions (F. Hoffmann-La Roche AG; Basel, Switzerland), with a color development incubation of 25 min at room temperature. Results are presented as a percentage of the dynamic range enclosed by the spontaneous LDH release designated as 100% and total LDH release (Triton X-100-treated controls (1% v/v for 2 h)) as 0%. Particle-only dose-dependent LDH activity interference is presented in Fig. S8; adjustment was performed as described in the Supplemental methods.

#### Post-treatment proliferative capacity

Beas-2B cells were plated in 6-well plates at a density of 50,000 cells per well. After 2 days, cells were treated with thermoplastics or B[a]P for 3 days, the cells washed once with DPBS, and then retreated for an additional 2 days prior to trypsinization and replating in 96-well microplates at a density of 1000 cells/well to quantitate doubling time using WST1. WST1 optical density values were used to derive the doubling time in log-phase growth using the package “growthcurves” in the statistical program R version 3.6 (R Project for Statistical Programming; Vienna, Austria).

#### Clonogenic assay

Beas-2B cells were plated in 6 well plates at an initial density of 300 cells per well [99]. The medium was refreshed with incinerated thermoplastic-containing AEGM, and 0.5 μM B[a]P or DMSO vehicle as control treatments. The cells were gently washed once with growth medium on day three and held in growth medium for an additional 7–10 days, or until individual colonies were apparent. Cells were then fixed with 4% paraformaldehyde for 15 min, and stained with 0.5% w/v crystal violet, followed by destaining in dH_2_O and imaging.

#### Live cell imaging

Propidium iodide (PI) is known to be excluded from membrane-intact cells and was used in combination with raw cell counts as a proxy for cellular cytotoxicity. After a 24- or 48-h exposure, thermoplastic-containing medium was replaced with fresh AEGM supplemented with 1 μM Hoechst 33342 (Thermo Fisher) and 5 μg/mL PI (Thermo Fisher), and imaged using the ImageXpress Micro XLS with MetaXpress software v.6 (Molecular Devices). Nuclei were visualized using a standard DAPI filter set, while necrotic cells were visualized using a standard Cy5 filter set. Hoechst 33342-reported cell number for each treatment group was normalized against untreated controls, and the remaining population was dichotomously assigned viable (PI negative) or necrotic (PI positive) based on Triton X-100-treated cells as intensity-based gating controls for PI positivity scoring. The integrated viability estimate adjusted for cell number (PI-) captures lytic cytotoxicity as well as reduction in proliferation. Results are expressed as percent PI- viable cells.

#### Mitochondrial membrane potential (ΔΨm)

ΔΨm was assessed ratiometrically using JC-1 (5,5′,6,6′-tetrachloro-1,1′,3,3′-tetraethyl-benzimidazolylcarbocyanine iodide) dye. Beas-2B or pSAECs were plated in clear-bottom, black-walled 96-well microplates and treated with incinerated thermoplastics as described above. After 24-h treatments, growth medium was replaced with fresh medium containing 1 μg/mL of JC-1 (ThermoFisher Scientific) and 1 μM Hoechst 33342 for 15 min. Cells were washed twice with fresh, pre-warmed growth media prior to assessment. Ten micrometre valinomycin for 30 min immediately after JC-1 staining served as a ΔΨm dissipation control. J-aggregates and monomers of JC-1 were quantitated at excitation/emission wavelength sets of 535/590 nm and 485/530 nm, respectively, using the SpectraMax M4 multimodal plate reader (Molecular Devices). Data were acquired using the SoftMax Pro v. 6.2.1 data reduction software (Molecular Devices). Using the ImageXpress high-content imager nuclei were visualized using a standard DAPI filter set, while J-aggregates and monomers were visualized using TRITC and FITC filters, respectively. An identification mask was generated based on nuclear Hoechst 33342 intensity and overlaid onto each nucleus for single cell identification. Similar masks were applied to TRITC and FITC channels based on dye intensity using the MetaXpress v.6 Software (Molecular Devices) prior to single-cell analysis in R. Similar to analysis using the microplate reader, ΔΨm was determined using average cell TRITC-to-FITC ratios.

#### Proliferation and nuclear morphometry

5-Ethynyl-2′-deoxyuridine (EdU) incorporation in actively proliferating cells [100] was performed using the Click-It EdU 647 Imaging Kit according to manufacturer’s instructions (ThermoFisher Scientific). Briefly, Beas-2B cells plated at 2500 cells per well in 96 well plates 2 days prior to exposure. Cells were then treated with incinerated thermoplastics for 24 h prior to incubation with 10 μM of EdU reagent in fresh complete culture medium for 60 min. Incorporated EdU was detected with an Alexa 647-conjugated azide as per manufacturer’s instructions (Thermo-Fisher), followed by probing with phospho-Histone H3 (1:400) and Ki-67 (1:400) in 1% BSA in D-PBS for 1 h. Phospho-Histone H3 and Ki-67 were then labeled with Alexa-555- and Alexa-488-conjugated F(ab’)_2_ secondary antibodies, respectively, and nuclei counterstained with 1 μM Hoechst 33342. Images were acquired at 20X using the ImageXpress. An identification mask was overlaid onto each nucleus, and morphometric parameters, including (p)-Histone H3, EdU, and Ki-67 positivity, nuclear area, nuclear Hoechst 33342 intensity, and EdU intensity were quantitated using the MetaXpress v.6 Software (Molecular Devices) prior to single-cell analysis in R. Cell cycle designations were assessed via a combined label-free method [101] with refinement using specific markers for proliferation/cell phase delineation [102]. Cells categorized into G1 were designated as Ki-67^+^/EdU^−^ with a nuclear intensity less than the median of EdU^+^ cells. Cells categorized into G2 were designated as Ki-67^+^/phospho-Histone H3^Ser10−^/EdU^−^ with a nuclear intensity greater than the median of EdU^+^ cells. G0 cells were designated as any cell Ki-67^−^. pSAEC particle uptake resulted in non-specific binding of EdU and antibodies to intracellularly endocytosed particles, thus invalidating the method for this model.

### ROS measurement

Beas-2B or pSAECs were plated with 5000 and 7500 cells per well, respectively, in clear-bottom, black-walled 96-well microplates and cultured 2 days prior to treatment with incinerated thermoplastics. At designated time points, thermoplastic- and menadione-treated cells were stained with 5 μM of CellROX Green (ThermoFisher Scientific) and 1 μM Hoechst 33342 in complete medium for 30 min under standard culture conditions and visualized using the ImageXpress. Nuclei were visualized using a standard DAPI filter set, while CellROX was visualized using a FITC filter set. Treatment with 100 μM menadione (MP Biomedicals, LLC.; Solon, OH) for 60 min in complete growth medium was used as a positive ROS control prior to ROS assessment. After measurement/imaging, cells were fixed using 4% formaldehyde for 15 min at room temperature. A binary mask was generated based on nuclear Hoechst 33342 intensity from the DAPI channel and overlaid onto each nucleus for single cell identification. An intensity-based mask for the FITC channel was applied covering the nucleus and cytoplasm using the MetaXpress. Cell-specific masks were then quantitated for average intensity prior to comparisons.

### Genotoxicity

Nuclear γH2AX positivity serves as a marker of stress induced by genotoxic agents [103]. After CellROX Green imaging, cells were probed using a 1:250 rabbit α-γH2AX mAb (Cell Signaling Technology) in 1% BSA in DPBS for 1 h at room temperature. γH2AX localization was tagged using an Alexa Fluor 647-conjugated goat α-rabbit secondary antibody F(ab’)2 fragments (Cell Signaling Technology), and nuclei counterstained with Hoechst 33342. Wells were imaged using the ImageXpress. Five hundred micrometre H_2_O_2_-treated cells (2 h prior to fixation) served as a positive control for γH2AX formation. A binary mask was generated based on nuclear Hoechst 33342 intensity from the DAPI channel and overlaid onto each nucleus for single cell identification. An intensity-based mask for the Cy5 channel was applied covering the nucleus using the MetaXpress. Cell-specific masks were then quantitated for average intensity prior to comparisons.

### Aryl hydrocarbon receptor

#### Cytochrome P450 1 induction

Cytochrome P450 1 isoforms (CYP1A and B) were assessed using a luminescence-based activity assay as per manufacturer’s instructions (Promega Corporation; Madison, WI). Briefly, Beas-2B cells were plated in 96-well plates at a density of 5000 cells/well. 48 h post-treatment, cells were incubated with 100 μM luciferin-CEE for 3 h under standard culture conditions. Thereafter, an aliquot of cell culture supernatant was abstracted and incubated in an equal volume of luciferase detection reagent at RT and quantitated using the Varioskan LUX multimodal plate reader (Thermo Fisher) with a 1.5 s integration time – cell-free medium served as the assay blank. 0.5 μM B[a]P served as a control for CYP1 activity induction.

### Protein analysis

Beas-2B cells were plated in 12-well microplates at a density of 62,000 cells per well 48 h prior to treatment. Cells were then treated with 0.5 μM B[a]P for 24 h. Beas-2B cells were lysed in RIPA buffer supplemented with 1 mM PMSF (Millipore-Sigma), 1 mM Na-orthovanadate, and 1X proteinase inhibitor cocktail (Santa Cruz Biotechnologies; Dallas, TX). Whole cell lysate preparations were ultrasonicated for 5 s on ice, centrifuged at 13,000 x g for 10 min at 4 °C, and the supernatant moved to clean tubes for storage at − 80 °C. Lysate protein quantitation was performed via the BCA method.

Protein expression of whole cell lysates was evaluated using the ProteinSimple Wes (ProteinSimple; San Jose, CA) with total protein normalization as the loading control. Briefly, lysates were diluted to 1.0 μg/μL in 0.1X sample buffer supplied by ProteinSimple. All primary and secondary antibodies were purchased from Cell Signaling Technologies (Danvers, MA). Primary antibodies used to detect checkpoint induction were as followed: phospho-cdc2(Tyr15), phospho-Chk1(Ser345), which were diluted 1:50 in supplied antibody diluent. For chemiluminescent signal detection, primary antibodies were probed using a 1:100 anti-rabbit-HRP secondary antibody in supplied antibody diluent.

### Statistical analysis

Regression modeling and statistical analyses were carried out using the R statistical program. Statistical comparisons were performed via Student’s t-test. Dose-response modeling and associated ED_50_ derivations were attained using the “drc” package as described by Ritz et al. [104]. In the case of WST1, ED_50_ and ED_110_ values were derived from a modified non-linear five-parameter log-logistic (Cedergreen-Ritz-Streibig) model that accounts for hormetic response. Comparisons of ED_50_ were made via Student’s t-test. Growth curves for deriving doubling time were modeled using the “growthcurves” package in R. All point estimates are the arithmetic mean of independent experiments with error bars indicating one standard error of the mean.

## Supplementary information

**Additional file 1: Table S1.** Medium-specific physicochemical properties for DLS measurement. **Figure S1.** SEM and EDX of incinerated thermoplastics. **Figure S2.** Enhanced darkfield microscopy of particle-only suspensions. **Figure S3.** Dose-response in Beas-2B using administered dose as the exposure metric. **Figure S4.** Comparison of ΔΨm on two different analytical platforms. **Figure S4.** Comparison of ΔΨm on two different analytical platforms. **Figure S5.** iNEC-induced intracellular ROS in pSAECs. **Figure S6.** Particle interference with γH2AX assessment in pSAECs. **Figure S7.** Endotoxin content and adsorption of incinerated thermoplastics. **Figure S8.** Lactate dehydrogenase interference testing in the presence of thermoplastics.

**Additional file 2.** Extended description of the methods has been included with additional details employed in this manuscript.

## Data Availability

The data generated or analyzed during this study are included in this published article [and its supplementary information files] and are available from the corresponding author on reasonable request.

## Abbreviations

αNF: Alpha-naphthoflavone
ΔΨm: Mitochondrial membrane potential
AEGM: Airway epithelial growth medium
B[a]P: Benzo[a]pyrene
CYP1: Cytochrome P450, family 1
DG: Distorted Grid
DMSO: Dimethyl sulfoxide
CNT: Carbon nanotube
CytoD: Cytochalasin D
DEP: Diesel exhaust particle
DSE_cr_: Delivered critical sonication energy
EDM: Enhanced darkfield microscopy
EdU: 5-Ethynyl-2′-deoxyuridine
EDX: Energy dispersive x-ray spectroscopy
iNEC: Incinerated nano-enabled composite
LDH: Lactate dehydrogenase
MSW: Municipal solid waste
NEC: Nano-enabled composite
PAH: polycyclic aromatic hydrocarbon
PC: Polycarbonate
PC-CNT: Polycarbonate with 3% w/w carbon nanotube
PDI: Polydispersity index
PI: Propidium iodide
PI-: Propidium iodide positivity adjusted for cell number
pSAEC: Primary small airway epithelial cell
PU: Polyurethane
PU-CNT: polyurethane with 0.1% w/w carbon nanotube
ROS: Reactive oxygen species
SAGM: Small airway epithelial growth medium
SEM: Scanning electron microscopy
TEM: Transmission electron microscopy
VOC: Volatile organic chemical
