# Supplementary material for: Carbon nanotube filler enhances incinerated thermoplastics-induced cytotoxicity and metabolic disruption in vitro
Source: Part Fibre Toxicol. 2020 Aug 12;17:40. doi: 10.1186/s12989-020-00371-1 (PMC7424660; doi:10.1186/s12989-020-00371-1)
Supplement: Supplementary file 1 — Additional file 1: Table S1. Medium-specific physicochemical properties for DLS measurement. Figure S1. SEM and EDX of incinerated thermoplastics. Figure S2. Enhanced darkfield microscopy of particle-only suspensions. Figure S3. Dose-response in Beas-2B using administered dose as the exposure metric. Figure S4. Comparison of ΔΨm on two different analytical platforms. Figure S4. Comparison of ΔΨm on two different analytical platforms. Figure S5. iNEC-induced intracellular ROS in pSAECs. Figure S6. Particle interference with γH2AX assessment in pSAECs. Figure S7. Endotoxin content and adsorption of incinerated thermoplastics. Figure S8. Lactate dehydrogenase interference testing in the presence of thermoplastics. [file 12989_2020_371_MOESM1_ESM.docx]

Carbon Nanotube Filler Enhances Incinerated Thermoplastics-induced Cytotoxicity and Metabolic Disruption *In Vitro*

Jayme P. Coyle*^1^, Raymond C. Derk^1^, Tiffany G. Kornberg^1,2^, Dilpreet Singh^3^, Jake Jensen^3^, Sherri Friend^1^, Robert Mercer^1^, Todd A. Stueckle^1^, Philip Demokritou^3^, Yon Rojanasakul^2^, Liying W. Rojanasakul^1^

^1^HELD/ACIB, National Institute for Occupational Safety and Health, Morgantown, WV

^2^Department of Pharmaceutical Sciences, West Virginia University, Morgantown, WV

^3^Department of Environmental Health, Harvard University, Boston, MA

**Corresponding Author**

Jayme P. Coyle, Email: [nti2@cdc.gov](mailto:nti2@cdc.gov)

**Supplemental Table**

| **Table S1.** Medium-specific physicochemical properties for DLS measurement | | | | |
| --- | --- | --- | --- | --- |
| Medium | Density (g/cm^3^) | Refractive Index | Dynamic Viscosity (cP) | Permittivity (dyne/cm) |
| dH2O | 0.99336 | 1.333 | 0.708 | Varies |
| AEGM | 1.00425 | 1.336 | 0.765 | 78 |
| SAGM | 1.00452 | 1.336 | 0.768 | 78 |
| Measurements for each parameter were performed at 37 degrees Celsius, except for electrical permittivity. These values were assumed to be similar to a typical buffer containing high salt concentrations, such as phosphate buffered saline. | | | | |

**Supplemental Figures**


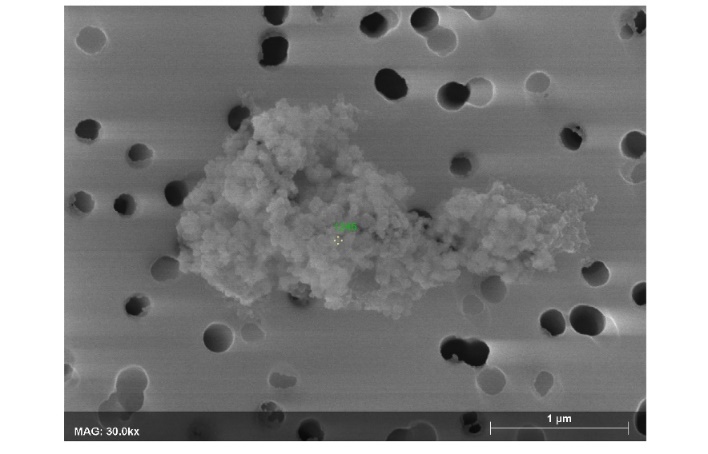

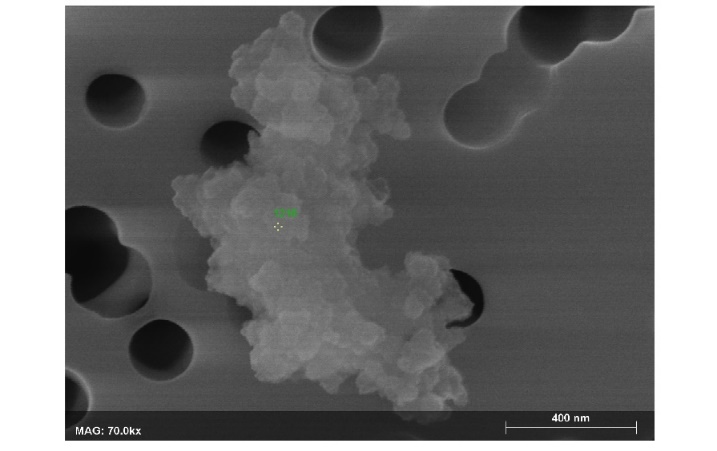

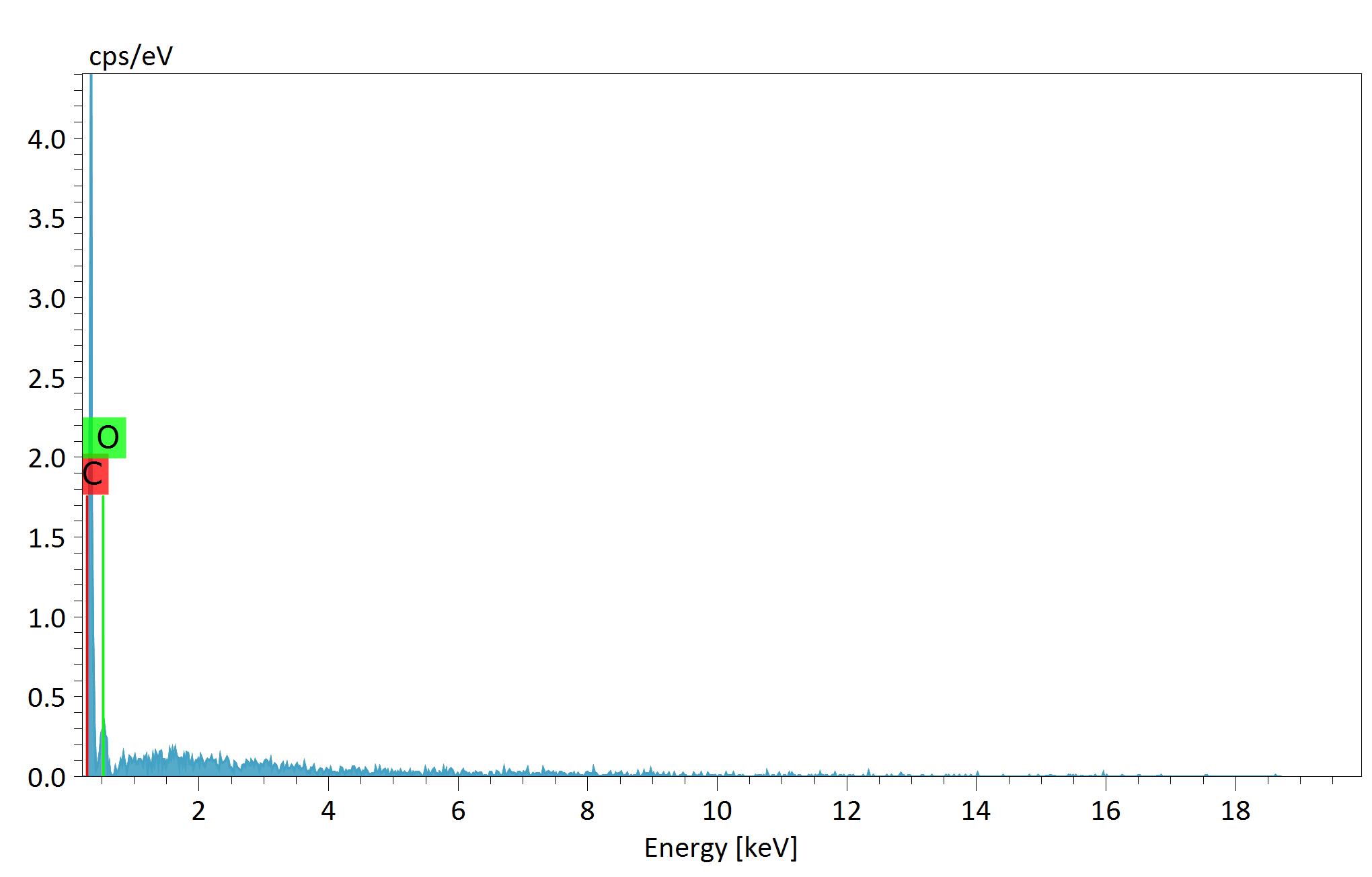

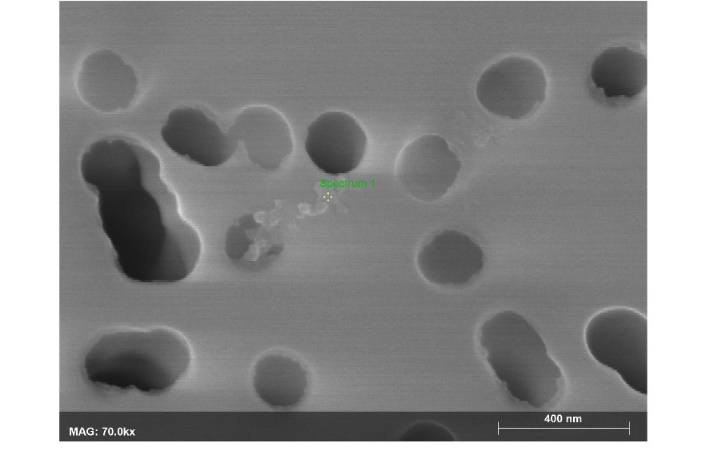

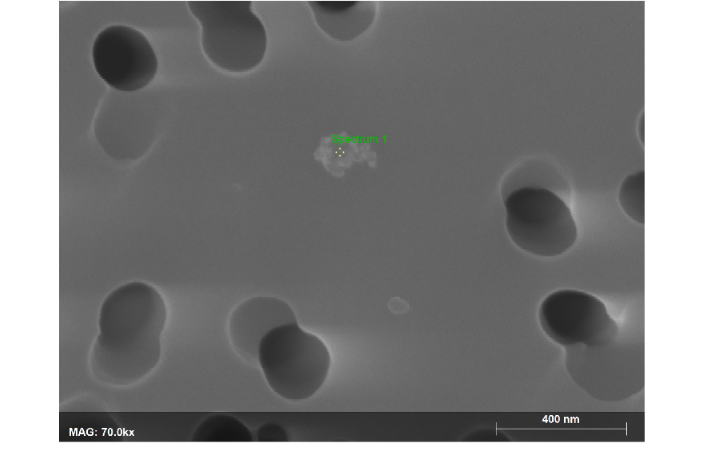

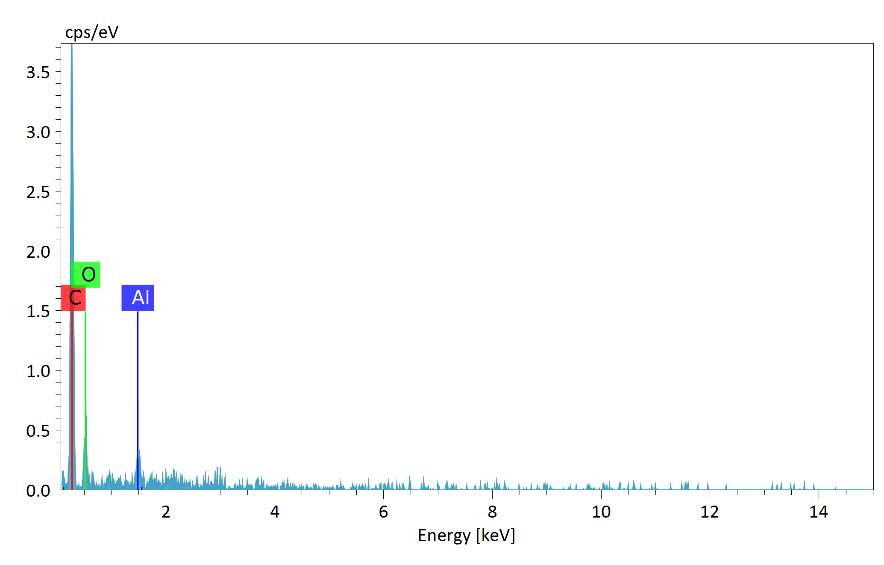

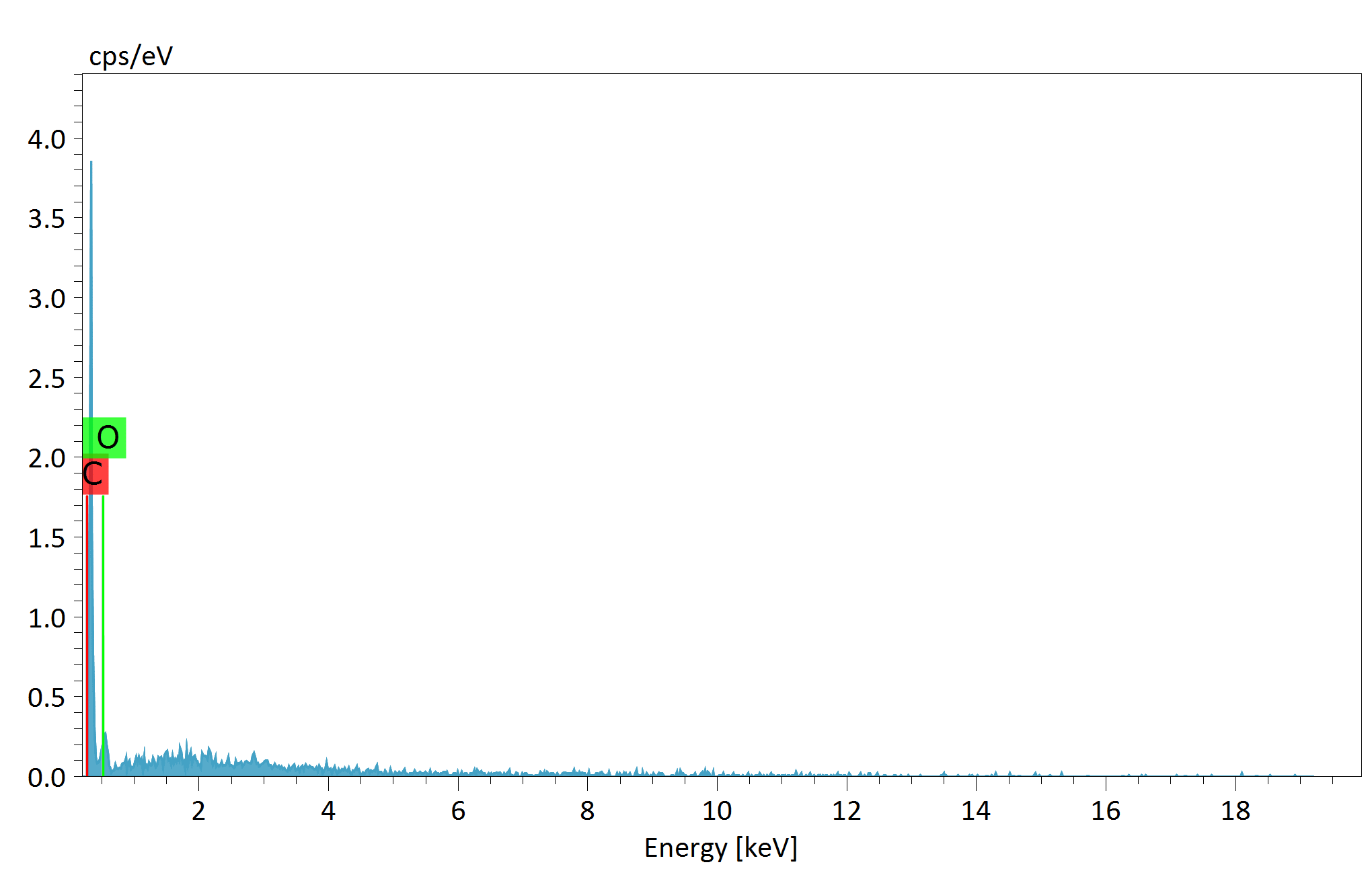

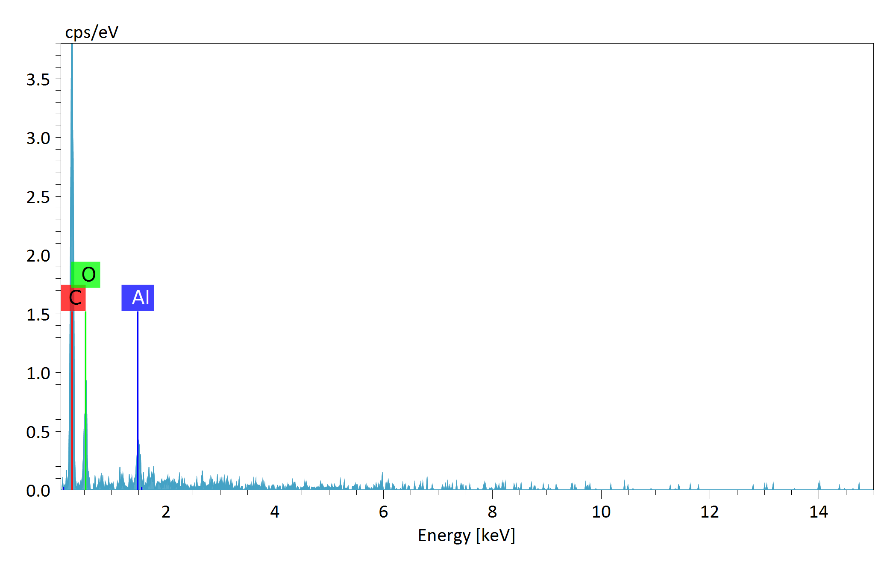


**PC-CNT**

**PC**

**PU**

**PU-CNT**


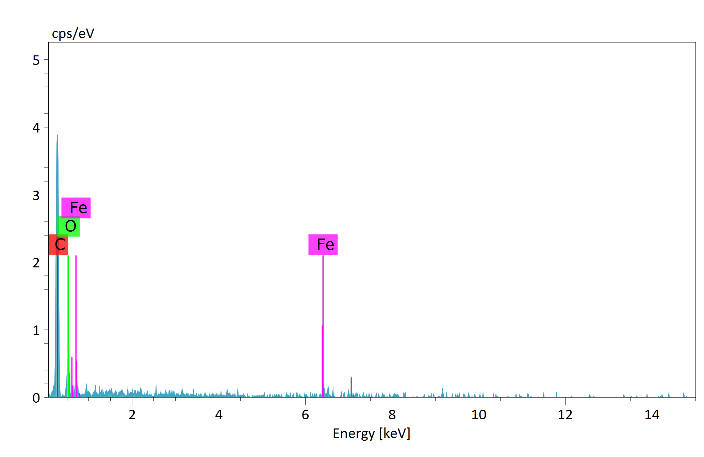

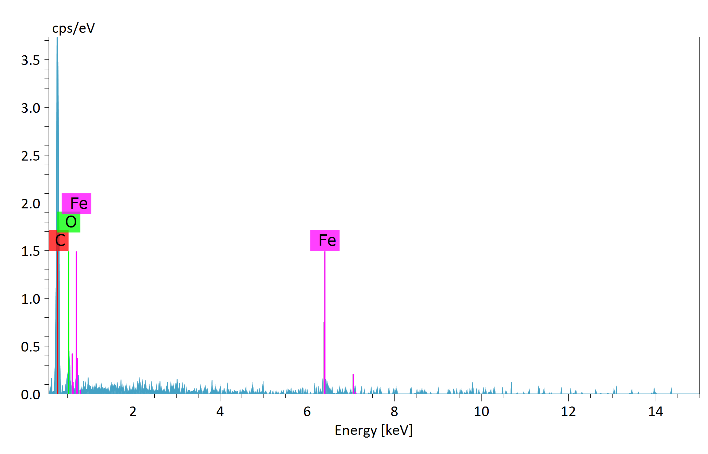


**Fig. S1**. SEM and EDX of incinerated thermoplastics. Thermoplastic preparations in water were assessed for elemental analysis using EDX in conjunction with SEM.

**Fig. S2**. Enhanced darkfield microscopy of particle-only suspensions. Incinerated thermoplastics visualized by EDM are identified by a bright spectral signature. PU/-CNT were more readily visualized compared to PC/-CNT analogues. Magnification: 600X, scale bar = 10 µm. (a) incinerated PC, (b) incinerated PC-CNT, (c) incinerated PU, (d) incinerated PU-CNT.

**Fig. S3**. Dose-response in Beas-2B using administered dose as the exposure metric. The data composing Figure 2a of the text was re-formatted such that administered dose was utilized as the exposure metric. Doses are presented on mass administered per volume (a) and mass administered per well surface area (b) basis. ED_50_ values were not derived as time-matched dose-response curves for PC and PC-CNT overlay substantially. Point estimates are the arithmetic mean of 3-4 independent experiments; error bars indicate standard error of the mean (SEM).

**Fig. S4**. Comparison of ΔΨm on two different analytical platforms. Beas-2B cells treated with incinerated thermoplastics in the manuscript were assessed for ΔΨm on two platforms: ImageXPress Micro XLS (HCS) and SpectraMax M4 Microplate Reader (MPR). HCS data are presented in Figure 2e. During testing, the ImageXPress was rendered inoperable, thus requiring use of the Microplate Reader platform for the data presented in Figure 3c. Point estimates are the arithmetic mean of 3 independent experiments; error bars indicate standard error of the mean (SEM); *p < 0.05, **p < 0.01, ***p < 0.001 compared to respective controls per t-test.

**Fig. S5**. iNEC-induced intracellular ROS in pSAECs. Intracellular ROS was measured 24 and 48 hours after treatment; 100 µM Menadione served as a positive control for ROS generation. Point estimates are the arithmetic mean of 2 independent experiments; error bars indicate standard error of the mean (SEM); **p < 0.01, compared to respective controls per t-test.


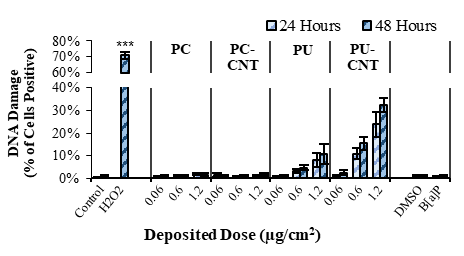


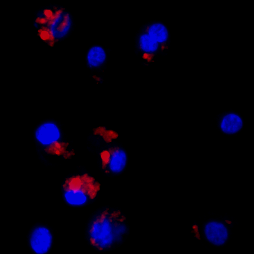

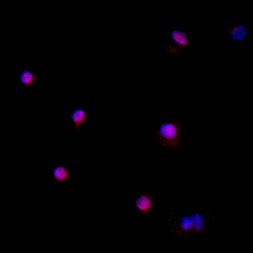

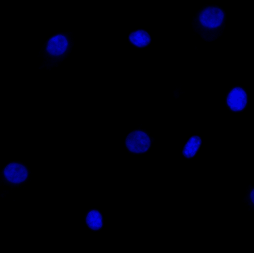

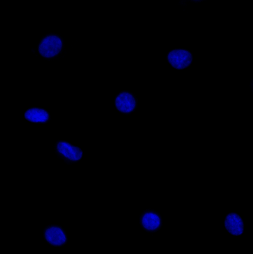


Control

H_2_O_2_

1.2 μg/cm^2^ PC-CNT

1.2 μg/cm^2^ PU-CNT

**Fig. S6**. Particle interference with γH2AX assessment in pSAECs. γH2AX assessment was conducted on pSAECs and quantitated for DNA γH2AX positivity. Treated pSAECs are exhibited large, high-intensity perinuclear staining among PU/PU-CNT-treated pSAECs which was associated with dose-dependent increases in γH2AX positivity. Qualitative examination of these perinuclear aggregate patterning against the spectral signatures observed from EDM concluded the fluorescence observed was non-specific. Point estimates are the arithmetic mean of 2 independent experiments; error bars indicate standard error of the mean (SEM). Statistical tests were not performed on cells treated with PU/PU-CNT due to significant interference with antibody staining, all other groups, except the positive control, were not significantly different than controls. ***p < 0.001 compared to respective controls.

**Fig. S7**. Endotoxin content and adsorption of incinerated thermoplastics. 10 µg/mL incinerated thermoplastics were suspended in endotoxin-free water. Endotoxin content was assessed for particles alone (Black Bars) or on particle suspensions spiked with 0.05 Endotoxin Units/mL (Red Bars). Proportion of spiked endotoxin adsorbed was quantitated from particle-free spike controls (0.05 EU/mL) is presented above respective Red Bar. Estimates are the arithmetic mean of two replicates; error bars represent SEM. LOQ = 0.01 EU/mL.

**Fig. S8**. Lactate dehydrogenase interference testing in the presence of thermoplastics. Purified LDH (2.5 mU/ Well) were incubated with graded depositional doses of incinerated thermoplastics in AEGM for 24 hours prior assessment. Data are normalized against the untreated 2.5 mU/well sample, arbitrarily designated as 0% Interference. As interference values tested were approximately linear, a simple linear regression curve was fitted for each individual particle, and the resulting regression coefficients used to correct LDH values among all deposited doses.
