# Supplementary material for: Carbon nanotube filler enhances incinerated thermoplastics-induced cytotoxicity and metabolic disruption in vitro
Source: Part Fibre Toxicol. 2020 Aug 12;17:40. doi: 10.1186/s12989-020-00371-1 (PMC7424660; doi:10.1186/s12989-020-00371-1)
Supplement: Supplementary file 2 — Additional file 2. Extended description of the methods has been included with additional details employed in this manuscript. [file 12989_2020_371_MOESM2_ESM.docx]

Carbon Nanotube Filler Enhances Incinerated Thermoplastics-induced Cytotoxicity and Metabolic Disruption *In Vitro*

Jayme P. Coyle*^1^, Raymond C. Derk^1^, Tiffany G. Kornberg^1,2^, Dilpreet Singh^3^, Jake Jensen^3^, Sherri Friend^1^, Robert Mercer^1^, Todd A. Stueckle^1^, Philip Demokritou^3^, Yon Rojanasakul^2^, Liying W. Rojanasakul^1^

^1^HELD/ACIB, National Institute for Occupational Safety and Health, Morgantown, WV

^2^Department of Pharmaceutical Sciences, West Virginia University, Morgantown, WV

^3^Department of Environmental Health, Harvard University, Boston, MA

**Corresponding Author**

Jayme P. Coyle, Email: nti2@cdc.gov

**Supplemental Methods**

Particle Characterization and Dosimetry

All incinerated pristine and nano-enabled thermoplastics used in this study were provided by P. Demokritou. Previous work using these materials characterized CNT loading and signature impurities^29,42^ pertinent to this work. Briefly, PC-CNT and PU-CNT contained respectively 3% and 0.1% MWCNT loading by weight, as disclosed in Singh *et al.*^29^

Particle deposition for thermoplastics was modeled using the Harvard Distorted Grid model as described in DeLoid *et al.*^89^ to estimate deposited dose up to 72 hours post-treatment. As modeling input parameters, the medium density, viscosity, and refractive index of complete cell growth media were measured and used, as well as the effective density of each incinerated thermoplastic in respective medium. Deposited modeling was conducted using MatLab (MathWorks, Inc., Natick, MA). With the exception of the data derived from dynamic light scattering and deposited modeling, medium density, viscosity and refractive index may be found within Supplemental Table T1 provided.

*Medium Density, Viscosity, and Refractive Index.* For all measurements, media were first equilibrated to room temperature. Media density was measured using a tared 10 mL volumetric flask and an analytical balance. Each medium was measured in quintuplicate. Viscosity was assessed using a size 25 Cannon-Fenske Routine viscometer (Cannon Instruments; State College, PA). After media equilibration, the viscometer was filled, and the body submerged in water maintained at 37 °C for a minimum of 10 minutes prior to measurement and calculation of kinematic viscosity. The kinematic viscosity was then used to calculate the dynamic viscosity necessary for dosimetry modeling. Refractive index was determined using an ISO-9001 certified hand-held refractometer according to manufacturer’s instructions (Catalog: AR200; Reichert Analytical Instruments; Dewpew, NY). All media were equilibrated to room temperature, and analysis performed on a volume sufficient to coat the prism surface.

*Effective Density*. To assess effective density via the volumetric centrifugation method, incinerated thermoplastics were diluted to 0.1 mg/mL in respective medium and 1 mL loaded in into packed cell volume tubes (Cat. 87007, Techno Plastic Products, A.G., Trasadigen, Switzerland). Tubes were centrifuged at 3,000 x g for 1 hour at room temperature. The compacted pellet volume was manually measured using a PCV tube ruler (Cat. 87010 Techno Plastic Products, A.G.). The density, packed pellet volume, medium density were used to calculate the effective density (*ρ_EV_*) requisite for modeling.

*Hydrodynamic Diameter by Dynamic Light Scattering*. The critical delivered sonication energy (DSE_cr_), which is the energy necessary to disperse a 1 mg/mL particle suspension in dH_2_O, for these particles was derived previously as 1066 J/mL for all thermoplastics.^41^ Particle suspensions were sonicated at the appropriate frequency and duration to attain the DSE_cr_ using a Cup Horn Sonicator (Sonics VibraCell VCX-750 with Cup-type Sonicator; Newton, CT) immediately prior to dilution to 0.1 mg/mL in dH_2_O or culture medium. A Zetasizer Nano ZS equipped with the 633 nm laser and DipCell for electrophoretic measurements (Malvern Instruments; Malvern, United Kingdom), were used to evaluate hydrodynamic diameter, zeta potential, and conductivity of particle suspensions. Suspension pH was measured with a standard pH meter (Accumet Model 50, Fisher Scientific).

*Endotoxin Content*. All thermoplastics were screened for endotoxin content at a suspension concentration of 10 µg/mL in autoclaved water, and were found to be below the detection limit of < 0.01 EU/mL via the LAL chromogenic method (Pierce; A39553). In assessing endotoxin adsorption 10 µg/mL particle suspensions were spiked with 0.05 EU/mL, revealed in bound endotoxin ranging from 0% to 23% (Supplemental Figure S1), depending on the thermoplastic.

Cell Culture and Treatment

Human bronchial epithelial (Beas-2B) cells were purchased from ATCC (CRL-9609, Manassas, VA), and cultured in complete airway epithelial growth medium (AEGM) purchased from PromoCell, GmbH (Heidelberg, Germany). Human primary small airway epithelial cells (pSAECs) were purchased from PromoCell and maintained in small airway epithelial growth medium (SAEGM) from the same vendor. When 70-80% confluent, cells were washed once with HEPES buffered saline prior to trypsinization. Trypsin was neutralized using soybean trypsin neutralization solution (PromoCell), and separated from the cells by light centrifugation (220 x g, 3 minutes) before being counted (Invitrogen Countess, Fisher Scientific) and re-plated.

Both cell types were seeded into microplates at subconfluency as described for individual assays below. The culture medium was aspirated and replaced with fresh pre-warmed medium containing treatment particle at designated delivered doses, calculated as described above. 1.0 mg/mL stock solutions of all test particles were sonicated to a dispersion energy of 1,066 J/mL immediately before dilution in growth medium. All dosing designations are reported as deposited dose (µg/cm^2^). For indicated experiments, cells were pre-incubated with named inhibitors: cytochalasin D (CytoD; Millipore-Sigma), CH333191 (Millipore-Sigma; Cat. C8124), or alpha-naphoflavone (αNF; Millipore-Sigma), for one hour before the incinerated thermoplastic treatments, and continued for the duration of incinerated thermoplastic exposure.

Microscopy

To evaluate particle uptake in SAECs and Beas-2B cells, two methods of microscopic analysis were employed.

*Enhanced Darkfield Microscopy*. Cells were seeded onto round laser-cut glass coverslips (Schott, A.G.; Jena, Germany) within 6-well tissue culture-treated microplates at a density of 150,000 cells per well. Two days later, cells were treated with 0.6 µg/cm^2^ of each thermoplastic in complete medium for 48 hours, fixed in 4% formaldehyde in DPBS, and mounted on slides. Coverslips were then sealed and imaged at 60X using the CytoViva EDM system (CytoViva, Inc.; Auburn, AL).

*Electron Microscopy*. For electron microscopy, cells were seeded at a density of 150,000 cells per well in tissue culture-treated 6-well microplates. Cells were then treated for 48 hours with 1.2 µg/cm^2^ incinerated thermoplastics, trypsinized with 0.25% Trypsin EDTA, washed twice with D-PBS, and fixed in Karnovsky’s fixative overnight as a single cell suspension. Pelleted cells were embedded in 4% agarose and returned to Karnovsky’s fixative overnight. After 3 buffer rinses, the cells were post-fixed in 1% osmium tetroxide, mordanted by 1% tannic acid and en-bloc stained with 0.5% uranyl acetate. A graded series of ethanol (50, 70, 90 & 100%) were used for dehydration. Propylene oxide served as an infiltrating agent before embedding in LX-112 epon. Blocks were cut at 70 nm, and the ultra-thin sections were placed on 200 mesh copper grids. Grids were stained using 4% uranyl acetate (nuclear stain) and Reynold’s lead citrate (cytoplasmic stain). Samples were imaged using a JEOL 1400 (Tokyo, Japan).

Particle only preparations were used to facilitate identification of endocytized particle, and were visualized using both SEM and TEM. For SEM, particles were diluted 1:10 in filtered distilled water. 0.5 ml of the particle solution was vacuumed filtered onto a 0.2 micron polycarbonate Millipore filter. The sample was sputter coated with gold-palladium for 2 minutes. Particles were imaged using a Hitachi S4800 field-emission scanning electron microscope (Tokyo, Japan). Energy dispersive x-ray generated qualitative elemental analysis (Bruker Nano, Berlin, Germany). For TEM, particles were diluted 1:10 in filtered distilled water. Using a tuberculin syringe, one drop was placed on a formvar coated 200 mesh copper grid, and evaporated over night. The samples were imaged using a JEOL 1400 transmission electron microscope (Tokyo, Japan).

Toxicological Analyses

*Cytotoxicity/Proliferation Assessment.* Five measures were employed to describe cytotoxicity and proliferative capacity: WST, LDH, clonogenic assay, live cell imaging, and mitochondrial membrane potential. Except for the clonogenic assay, Beas-2B and pSAECs were plated at a density of 5,000 cells and 2,500 cells per well, respectively, in 96-well microplates 2 days prior to exposure. For the post-treatment proliferative capacity, cells were plated in 6-well microplates at a density of 50,000 cells per well 2 days prior to treatment. Benzo[a]pyrene (0.5 µM) served as a PAH control and was purchased from Millipore-Sigma (Cat. 51968). All colorimetric endpoints were acquired using the SpectraMAX Plus 384 (Molecular Devices, Le Jolla, California) and the SoftMax Pro v. 5.4.1 data acquisition software.

*WST1 Tetrazolium Reduction*. Cells were treated in growth medium for 24-48 hours prior to assessment with WST1 (Cat. 11644807001; Millipore-Sigma, St. Louis, MO). After incinerated thermoplastic exposure, well contents were gently mixed, the plate centrifuged at 1,000 x rpm at RT for 5 minutes to remove cell debris, and an aliquot of supernatant abstracted into a clean clear-bottom microplate for LDH assessment. The culture medium was replaced with fresh medium, and the WST1 was performed according to the manufacturer’s instruction with a 2-hour incubation period. WST1 formazan was quantitated at 450 nm with a reference wavelength of 650 nm. Treatment groups were normalized against medium-only controls, which were arbitrarily denoted as 1. Cells treated with 1% Triton X-100 accompanied all tests as a control for 100% cytotoxicity.

*Lactate Dehydrogenase Activity*. The LDH assay was performed according to manufacturer’s instructions (LDH Cytotoxicity Detection Kit; Cat. 11644793001; Millipore-Sigma), with a color development incubation of 25 minutes at room temperature. Results are presented as viability with non-treated controls designated as 100% and Triton X-100-treated controls (1% v/v for 2 hours) as 0%; LDH-reported cytotoxicity was assumed to be linearly within this range. To assess thermoplastic interference with the LDH assay, purified human LDH-A (Cat. SAE0049, Millipore-Sigma) was diluted to 2.5 mU/mL in the presence of graded concentrations of each thermoplastic in 96-well TCT microplates. After a 24-hour incubation, LDH was assessed analogously to samples of treated Beas-2B or pSAECs. As is routinely problematic for nanoparticles, all incinerated thermoplastics were found to interfere with the method for assessing LDH activity, as indicated by direct analyte interference testing using purified LDH (Supplemental Figure). Within the tested range, LDH interference was approximately linear in relation to dose. Hence, the LDH results were adjusted proportionally based on linear regression derived from individual thermoplastic concentration-LDH activity interference values, thus yielding an adjusted cytotoxicity value. The data are presented as percent inhibition compared to the LDH standard 2.5 mU/mL LDH arbitrarily designated as the 0% inhibition value; all other samples were proportionally normalized to the LDH-only standard.

*Post-treatment proliferative Capacity*. Beas-2B cells were plated in 6-well plates at a density of 50,000 cells per well. After two days, cells were treated with thermoplastics for 3 days, the cells washed once with DPBS, and then treated for an additional two days prior to trypsinization and replating in 96-well microplates at a density of 1,000 cells/well to quantitate doubling time. Beas-2Bs were analyzed for proliferation via WST1 at 2 hours post-plating, and every 24 hours thereafter for 5 days. Blanked WST1 absorbance values were then used to derive doubling time using the package “growthcurves” in R.

*Clonogenic Assay*. Beas-2B cells were plated in 6 well plates at an initial density of 300 cells per well.^91^ Between 6- and 8-hours post-plating, the medium was removed and replaced with incinerated thermoplastic-containing AEGM. 0.5 µM B[a]P or DMSO vehicle-treated Beas-2B cells accompanied thermoplastic treatments. The cells were gently washed once with growth medium on day three and fed with fresh medium for an additional 7-10 days, or until individual colonies were apparent. Thereupon, the cells were fixed with 4% paraformaldehyde for 15 minutes, and stained with 0.5% w/v crystal violet, followed by dH_2_O destaining and imaging.

*Live Cell Imaging -* Propidium iodide (PI) is known to be excluded from membrane-intact cells and was, therefore, used in combination with raw cell counts as a proxy for cellular cytotoxicity. After exposure, thermoplastic-containing medium was aspirated and replaced with fresh medium supplemented with 1 µM Hoechst 33342 (Thermo Fisher) and 5 µg/mL PI (Thermo Fisher) and imaged using the ImageXPress Micro XLS. Hoechst 33342-reported cell number for each treatment group was normalized against untreated controls, and the remaining population was dichotomously assigned viable (PI-) or dead (PI+) based on Triton X-100-treated cells as intensity-based gating controls for PI positivity scoring. Nuclei were visualized using a standard DAPI filter set, while necrotic cells were visualized using a standard Cy5 filter set. The integrated viability estimate adjusted for cell number (PI+-CN) captures lytic cytotoxicity as well as reduction in proliferation. Results are expressed as percent PI+-CN viable cells.

*Mitochondrial Membrane Potential (ΔΨm)*. ΔΨm was assessed ratiometrically using JC-1 (Cat. T3168, ThermoFisher Scientific). Beas-2B or pSAECs were plated in clear-bottom, black-walled 96-well microplates, and cultured until 80% confluent prior to treatment with incinerated thermoplastics. After 24-hour treatments, growth medium was replaced with fresh medium containing 1 µg/mL of JC-1 and 1 µM Hoechst 33342. Cells were incubated for 15 minutes under standard culture conditions, then washed twice with fresh, pre-warmed growth media, and subsequently held in 100 µL of fresh growth medium until assessment. As a positive control for ΔΨm dissipation, cells were treated with 10 µM valinomycin for 30 minutes immediately after JC-1 staining. J-aggregates and monomers of JC-1 were quantitated at excitation/emission wavelength sets of 535/590 nm and 485/530 nm, respectively. Data were acquired using the SoftMax Pro v. 6.2.1 data reduction software. For verification using the ImageXPress Micro XLS, nuclei were visualized using a standard DAPI filter set, while J-aggregates and monomers were visualized using TRITC and FITC filters, respectively.

*Proliferation and Nuclear Morphometry*. EdU incorporation in actively proliferating cells^92^ was performed using the Click-It EdU 647 Imaging Kit according to manufacturer’s instructions (ThermoFisher Scientific). Briefly, Beas-2B cells or pSAECs were plated at 2,500 cells per well in 96 well plates 2 days prior to exposure. Cells were then treated with incinerated thermoplastics for 24 hours prior to incubation with 10 µM of EdU reagent in fresh complete culture medium for 60 minutes. Thereafter, the cells were fixed in 4% paraformaldehyde and permeabilized with 0.25% v/v Triton X-100 for 15 minutes each at RT, followed by EdU detection with an Alexa 647-conjugated azide as per manufacturer’s instructions (Thermo-Fisher; Cat.: C10419). The cells were then washed three times with D-PBS containing 1% w/v BSA and probed for phospho-Histone H3 (1:400) and Ki-67 (1:400) in 1% BSA in D-PBS for 1 hour. Phospho-Histone H3 and Ki-67 were then respectively labeled with Alexa-555- and Alexa-488-conjugated F(ab’)_2_ secondary antibodies at concentrations of 1:1000 in 1% BSA-containing D-PBS; nuclei were counterstained with 1 µM Hoechst 33342 in D-PBS. Images were acquired at 20X using the ImageXPress HCS imager. An identification mask was overlaid onto each nucleus, and morphometric parameters, including (p)-Histone H3, EdU, and Ki-67 positivity, nuclear area, nuclear Hoechst 33342 intensity, and EdU intensity were quantitated using the MetaXPress v.6 Software (Molecular Devices) prior to single-cell analysis in R. Cell cycle designations were assessed via a label-free method similar to analyses by flow cytometry^93^ and specific markers for proliferation/cell phase delineation.^94^ Cells categorized into G1 were designated as Ki-67^+^/EdU^-^ with a nuclear intensity less than the median of EdU^+^ cells. Cells categorized into G2 were designated as Ki-67^+^/phospho-Histone H3^Ser10-^/EdU^-^ with a nuclear intensity greater than the median of EdU^+^ cells. G0 cells were designated as any cell Ki-67^-^.

ROS Measurement

CellROX Green was employed for characterizing ROS associated with thermoplastic treatment. Beas-2B or pSAECs were plated with 5,000 and 7,500 cells per well, respectively, in clear-bottom, black-walled 96-well microplates and cultured 2 days prior to treatment with incinerated thermoplastics.

*CellROX Green*. At designated time points, thermoplastic- and menadione-treated cells were stained with 5 µM of CellROX Green (Cat. C10444, ThermoFisher Scientific) and 1 µM Hoechst 33342 in complete medium for 30 minutes under standard culture conditions. After staining, cells were gently washed thrice with respective medium, and CellROX fluorescence was measured using the SpectraMAX M4 plate reader (excitation/emission wavelengths: 485/520 nm). The same cells were visualized using the ImageXPress Micro XLS. Nuclei were visualized using a standard DAPI filter set, while CellROX was visualized using a FITC filter. Treatment with 100 µM menadione (Cat. 102259; MP Biomedicals, LLC. Solon, OH) for 30 minutes in complete growth medium was used as a positive ROS control prior to ROS assessment. After measurement/imaging, cells were fixed using 4% formaldehyde for 15 minutes at room temperature protected from light. The fixative was aspirated, and the cells washed once and subsequently held in fresh DPBS until probing for *γH2AX* – a marker for genotoxicity.

Genotoxicity

*γH2AX*. Nuclear γH2AX positivity serves as a marker of stress induced by genotoxic agents.^95^ After CellROX Green imaging, cells were fixed 4% paraformaldehyde and permeabilized using 0.25% Triton X-100 for 15 minutes, followed by blocking in 1% bovine serum albumin (Cat. Millipore-Sigma) in DPBS without Ca^2+^/Mg^2+^ for 30 minutes. γH2AX was probed using 1:250 rabbit α-γH2AX mAb (Cat. 9718; Cell Signaling Technology) in 1% BSA in DPBS for 1 hour at room temperature. Wells were washed thrice with DPBS and probed using Alexa Fluor 647-conjugated goat α-rabbit secondary antibody F(ab’)2 fragments (Cat. 4414, Cell Signaling Technology) in 1% BSA in DPBS for 1 hour at room temperature. Nuclei were counterstained with Hoechst 33342. Wells were washed thrice with and subsequently held in DPBS prior to imaging using the ImageXPress Micro XLS. 500 µM H_2_O_2_-treated cells (2 hours) served as a positive control for γH2AX formation.

Aryl Hydrocarbon Receptor

*Cytochrome P450 1 Induction*. Cytochrome P450 1 isoforms (CYP1) was assessed using a luminescence-based activity assay as per Manufacturer’s instructions (Cat. V8752; Promega). Briefly, Beas-2B cells were plated in 96-well plates at a density of 5,000 cells/well 48 hours prior to treatment. 48 hours post-treatment, cells were washed briefly with fresh medium and incubated with 100 µM luciferin-CEE for 3 hours under standard culture conditions. Thereafter, 25 µL of cell culture supernatant was abstracted and incubated in an equal volume of luciferase detection reagent at RT with periodic gentle mixing. Luminescent signal was quantitated using the Varoiskan LUX multimodal plate reader (Thermo Fisher) with a 1.5 second integration time – cell-free medium served as the assay blank. Cells treated with 0.5 µM B[a]P served as a control for CYP1 activity induction.

Protein Analysis

Beas-2B cells were plated in 12-well microplates at a density of 62,000 cells per well 48 hours prior to treatment. Cells were then treated with 0.5 µM B[a]P for 4, 24, and 48 hours. At the designated time point, Beas-2B cells were lysed in RIPA buffer supplemented with 1 mM PMSF (Millipore-Sigma), 1 mM Na-orthovanadate, and 1X proteinase inhibitor cocktail (Santa Cruz Biotechnologies, CA). Whole cell lysate preparations were centrifuged at 13,000 x g for 10 minutes at 4 °C, and the supernatant moved to clean tubes for storage at 80 °C. An aliquot of each sample was diluted 1:10 in DPBS for protein quantitation via the BCA method. As a positive control for proliferative inhibition, cells were pre-treated each with 3 µg/mL aphidicolin (G1/S Block; Millipore-Sigma, Cat.: A0781) or 100 ng/mL nocodazole (G2/M Block; Millipore-Sigma, Cat.: M1404) for 18 hours prior to lysis.

Protein expression of whole cell lysates was evaluated using the ProteinSimple Wes (ProteinSimple, San Jose, CA) with total protein as the loading normalization method. Briefly, lysates were diluted to 1.0 µg/µL in 0.1X sample buffer supplied by ProteinSimple. All primary and secondary antibodies were purchased from Cell Signaling Technologies (Danvers, MA). Primary antibodies used are as followed: phospho-cdc2(Tyr15), phospho-Chk1(Ser345), which were diluted 1:50 in supplied antibody diluent. For chemiluminescent signal detection, primary antibodies were probed using a 1:100 anti-rabbit-HRP secondary antibody in supplied antibody diluent.
